# Influence of the Substituents on the Opening of Silylepoxy Alcohols: 5-*exo*-Cyclization towards Tetrahydrofurans vs. Unexpected Side Reaction Leading to Tetrahydropyrans

**DOI:** 10.3390/molecules26237386

**Published:** 2021-12-06

**Authors:** Carlos Díez-Poza, Asunción Barbero

**Affiliations:** Department of Organic Chemistry, Faculty of Science, Campus Miguel Delibes, University of Valladolid, 47011 Valladolid, Spain; solracdp@hotmail.com

**Keywords:** tetrahydropyrans, tetrahydrofurans, cyclization, epoxysilyl alcohols, side reaction, tandem process

## Abstract

The regioselective ring opening of epoxy alcohols is an effective method for the synthesis of different types of oxacycles. The 5-*exo* opening being preferred vs. the 6-*endo* mode, according to Baldwin rules, the use of silyl-substituted oxiranes has been reported as a possible method to favor the 6-*endo* cyclization. However, there is a need for a detailed study on the different factors (structural factors, catalyst nature or conditions) that influence this process. In this paper, the acid-catalyzed cyclization of epoxysilyl alcohols was studied, focusing on the effect of substituents and reaction conditions on the outcome of the process. Two types of heterocycles (tetrahydrofurans or tetrahydropyrans) were selectively obtained depending on the structure of the initial epoxysilyl alcohol. Interestingly, cyclization of hindered epoxysilyl alcohols mainly proceeds through an unexpected side reaction, which implies a previous isomerization to an aldehyde. A mechanistic proposal for the formation of the different products is presented.

## 1. Introduction

**Figure 1:**
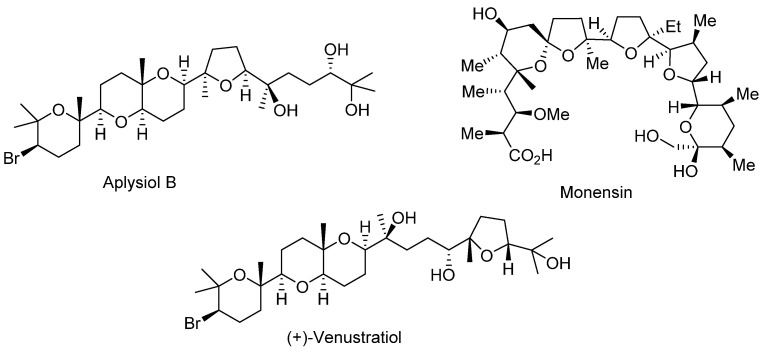
Polyether natural products.

Polyethers containing a set of interconnected oxacycles are found in a large number of naturally occurring products such as Monensin [1,2], which is an ionophoric antibiotic isolated from *Streptomyces cinnamonensis,* which shows various antimicrobial and biological activities; Venustatriol [3], a tetracyclic polyether isolated from red algae, which shows significant anti-viral activity; or Aplysiol B [4], which is a squalene-derived polyether isolated from the South China Sea hare *Aplysia dactylomela*, which shows ichthyotoxicity on G. affinis at a concentration of 10 ppm (Figure 1).

Due to the complex structural framework, great number of stereocenters and potential biological activities of these natural products, numerous research groups have been stimulated to study different synthetic approaches. Since tetrahydropyrans [5] and tetrahydrofurans [6,7] are the most common structural motifs in these polyheterocyclic compounds, considerable effort has been made towards the development of new routes to these substrates.

One common strategy to access the 5-membered or 6-membered oxacyclic structures is the acid-catalyzed intramolecular cyclization of epoxyalcohols [8]. Although Baldwin rules were not specifically set for the opening of epoxides, the literature shows that *exo* attack is usually favored vs. the *endo* attack.

**Scheme 1:**
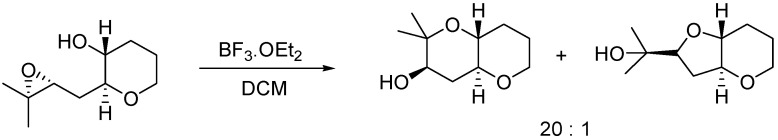
Alkyl directing 6-*endo* epoxide opening.

Many methodologies have been considered to control the regioselective outcome of the cyclization in one or the other sense. Since the *exo* attack is usually preferred, much effort has been made in the study of the factors that may promote the *endo* opening of the epoxide. For instance, alkyl-substituted epoxides have been employed [9] to favor the 6-*endo* cyclization, due to stabilization of the incipient tertiary carbocation in the transition state (Scheme 1).

**Scheme 2:**
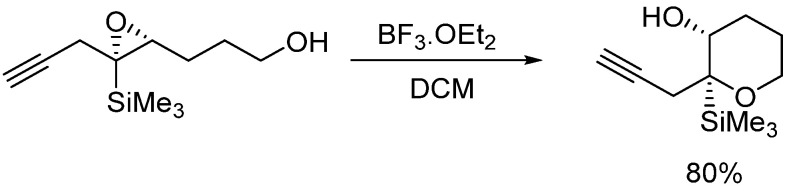
Silicon directing 6-*endo* epoxide opening.

On the other hand, Jamison’s group [10,11] has reported the directing effect of a silyl group bonded to the epoxide. The preferred intermolecular attack of nucleophiles to α,β-epoxysilanes, which proceeds with cleavage of the C-O bond α to the silicon atom, has been use to promote the *endo* opening of an epoxide which has a silyl group attached to the appropriate position (Scheme 2).

However, it has to be noted that, in these examples, the silyl group is attached to the more substituted carbon, which means that there may be two directing groups (the silyl and the alkyl groups) acting together. In fact, Schaumann has reported that the ring opening of 1,2-*trans*-disubstituted epoxides by a remote hydroxyl group provides mixtures of the 6-*endo* and 5-*exo* cyclization products [12].

During many years, our research group has been interested in the synthesis of carbo- [13,14] and heterocycles [15,16] using the chemistry of organosilanes. At this point, we thought that it would be interesting to study the independent effect of both silicon and alkyl groups in the acid-catalyzed cyclization of δ,γ-epoxy alcohols. In this sense, the use of our methodology of silylcupration of acetylenes and capture of the intermediate cuprate with α,β-unsaturated ketones will provide the perfect substrates for this study within two high-yielding steps.

## 2. Results and Discussion

**Scheme 3:**
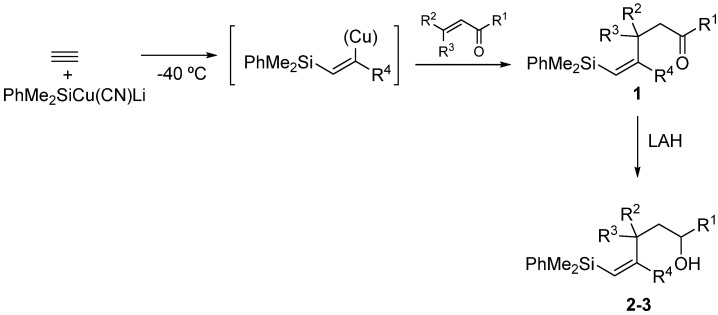
Synthesis of vinylsilyl alcohols **2-3**.

Silylcupration of acetylenes proceeds through a *syn*-addition of the pair Si-Cu to the triple bond, leading to a vinylsilane–vinylcopper intermediate that undergoes Michael addition in the presence of α,β-unsaturated ketones to give vinylsilyl ketones **1** in high yield [17].The reduction of these vinylsilyl ketones with LiAlH_4_ in anhydrous diethyl ether at 0 °C afforded, in quantitative yields, a diastereomeric mixture of the desired vinylsilyl alcohols **2-3**, which could be separated by chromatography (Scheme 3).

Compounds **2-3** were then treated with MCPBA at 0 °C for 2 h, providing either the corresponding epoxysilyl alcohols **4** or oxacycles **5-6**, formed by a domino epoxidation-acid catalyzed cyclization process. The results are shown in Table 1.

**Figure 2:**
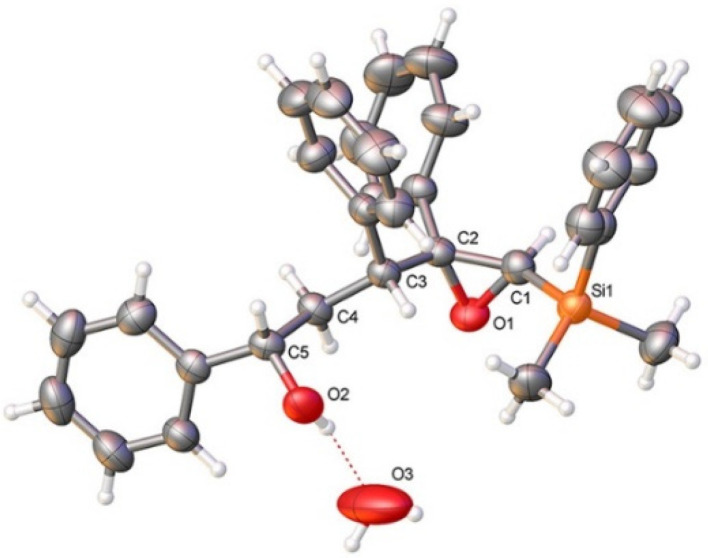
X-ray crystal structure of **4e**; displacement ellipsoids at 50% probability. Colour key: C (grey), N (blue), O (red), Si (orange). CCDC: 2118173.

As shown in Table 1, the reaction of vinylsilyl alcohols **2a-c** (R^4^ = H) with MCPBA undergo a tandem reaction which implies the epoxidation of the vinylsilane to give an epoxysilyl alcohol intermediate and subsequent acid catalyzed cyclization to provide tetrahydrofurans **5-6a-c**. On the other hand, the reaction with MCPBA of vinylsilyl alcohols **2d-e** and **3d**, with an extra substituent β to silicon, furnishes the expected epoxysilyl alcohols in good yields. It has to be noted that this epoxidation reaction proceeds with high stereocontrol, providing in most cases a single diastereoisomer, which corresponds with the epoxidation from the least hindered face of the alkene (opposite to the allylic substituent). The stereochemistry of the epoxysilyl alcohols could be unambiguously confirmed by X-ray diffraction analysis of compound **4e** [18] (Figure 2).

From the results shown in Table 1, we could draw the following conclusions: the tandem epoxidation-cyclization process is favored when the starting vinylsilyl alcohol lacks bulky substituents on the double bond, which seems to indicate that the cyclization process is slow for hindered epoxides. On the other hand, the acid-catalyzed ring closure of the intermediate epoxysilyl alcohols selectively furnishes the 5-*exo*-cyclization products, which correspond to the favored ring closure according to Baldwin’s rules. Remarkably, the reaction is completely regioselective and none of the regioisomeric tetrahydropyrans were observed in the reaction mixtures.

**Scheme 4:**
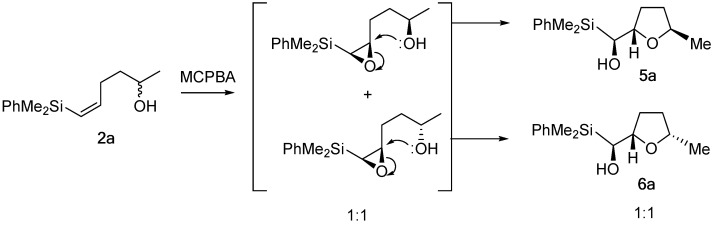
Stereoselective outcome of the domino epoxidation-cyclization process.

Regarding the stereoselective outcome of the domino epoxidation-cyclization, we could see two different behaviors. Thus, the domino process of substrates that have no allylic substituents furnish an equimolar mixture of the two C-5 epimeric THF. This result seems to indicate that the hydroxyl group is too far away from the vinylic moiety to exert any stereocontrol in the epoxidation step, with the epoxidation occurring from both faces of the alkene. However, the subsequent cyclization proceeds with total stereoselectivity, leading now to a unique isomer (Scheme 4).

On the other hand, vinylsilyl alcohols that bear an allylic substituent lead to a unique diastereomeric tetrahydrofuran. This fact corroborates our previous hypothesis, since now the presence of a bulky group next to the double bond exerts stereocontrol in the epoxidation step.

With these results in hand, we next decided to study the acid catalyzed cyclization of hindered epoxysilyl alcohols. For that purpose, we chose compound **4e** as a model and screened its reaction with different Lewis and Bronsted acids. The results are shown in Table 2.

**Scheme 5:**
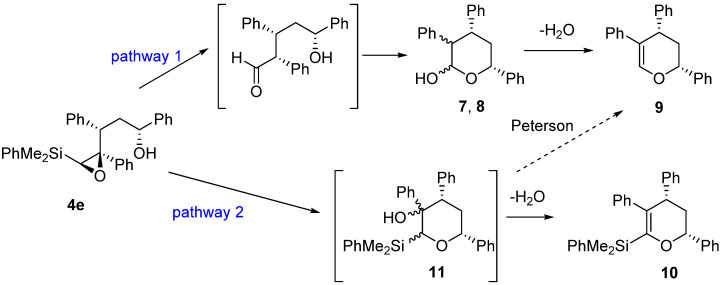
Mechanistic proposal for the formation of products from the acid-catalyzed cyclization of **4e**.

As shown, the acid-catalyzed cyclization of epoxysilyl alcohol **4e** affords up to three different types of products, from which tetrahydropyran **9** is the major one in most cases. Our mechanistic proposal (Scheme 5) for the formation of these products implies two different reaction pathways. Thus, the formation of hemiacetals **7**-**8** could be explained by an initial acid catalyzed isomerization of epoxysilane **4e** to the corresponding aldehyde, which cyclization would furnish hemiacetals **7**-**8**. The subsequent dehydration of these hemiacetals would provide the more stable dihydropyran **9**. On the other hand, the formation of **10** could be explained by an initial acid-catalyzed 6-*endo* cyclization of silylepoxy alcohol **4e**, to provide intermediate **11** (not observed), from which **10** would be obtained through a dehydration process. It has to be noted, that the Peterson elimination of **11** would also lead to compound **9** (pathway 2). However, since compound **10** is only observed (as a very minor subproduct) in one of the processes (Table 2, entry 1), we assume that compound **9** is formed through pathway 1, which seems to be the most favorable process from both pathways (Scheme 5).

We first studied the chemoselectivity of this process using different Bronsted and Lewis acids in dichloromethane at 0 °C (Table 2, entries 1, 3 and 5). As can be seen, the reaction in the presence of Bronsted acids provided dihydropyran **9** as the major product, along with a small amount of the hemiacetal **8** (Table 2, entries 1 and 3). It has to be noted that the reaction in neutral water, although substantially slower, provided a similar result to the use of CSA (Table 2, entries 3 and 4). Interestingly, the use at 0 °C of BF_3_.OEt_2_ as Lewis acid (Table 2, entry 5) provided an almost equimolar mixture of hemiacetal **7** and dihydropyran **9**.

We next decided to evaluate the effect of temperature in these processes. For that purpose, we chose BF_3_.OEt_2_ as acid. Enhanced chemoselectivity towards the formation of hemiacetal derivatives is observed when the temperature decreases, being able to suppress the formation of **9** when the reaction is conducted at either −40 °C or −78 °C. However, the reaction yield decreases significantly in those two examples (Table 2, entries 6 and 7).

**Figure 3:**
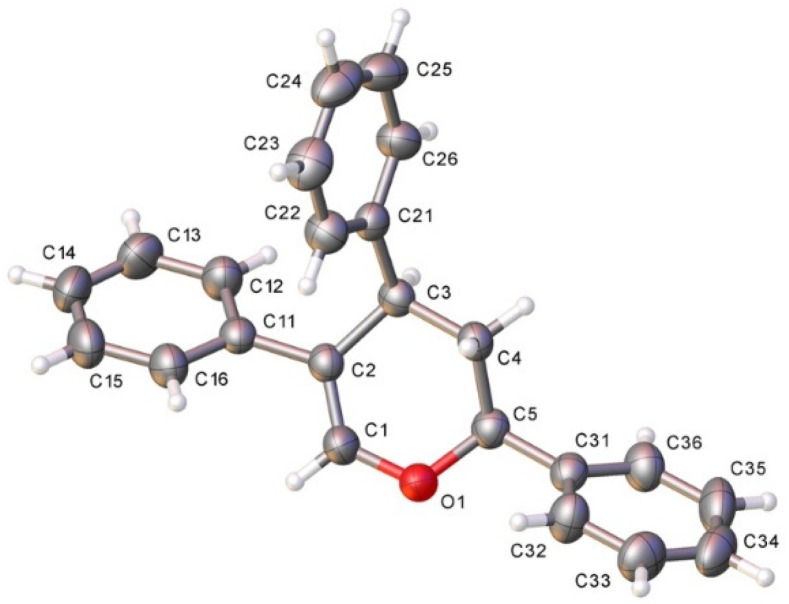
X-ray crystal structure of **9**; displacement ellipsoids at 50% probability. Colour key: C (grey), N (blue), O (red), Si (orange). CCDC: 2118174 [19].

The structure of dihydopyran **9** could be unambiguously confirmed by X-ray diffraction analysis (Figure 3).

## 3. Materials and Methods

### 3.1. General Procedures

Unless otherwise noted, all reagents were obtained from commercial suppliers as reagent grade and used without further purification. Compounds **1**-**3** were prepared according to literature [20].

Tetrahydrofuran, diethyl ether and dichloromethane were dried by standard methods (dichloromethane was freshly distilled from CaH_2_, tetrahydrofuran and diethyl ether were dried with preactivated molecular sieves (3Å, 1/16 in 1–2 mm diameter pellets). All experiments were performed in oven-dried glassware under an atmosphere of nitrogen using standard syringe techniques, except where otherwise noted. For all general procedures, the order of addition of reagents has to be respected. Low reaction temperatures were maintained using a Dewar System and an acetone bath cooled by liquid nitrogen. Yields refer to chromatographically and spectroscopically pure compounds, unless otherwise stated.

Flash column chromatography was performed using Silica Gel 60 (230–400 mesh ASTM) and ACS grade solvents. All product mixtures were analyzed by analytical thin-layer chromatography (TLC) using aluminium-backed plates, pre-coated with silica gel (0.20 mm, silica gel 60) with a fluorescent indicator (254 nm) from Macherey-Nagel and were performed using ACS grade solvents. Compounds were visualized by standard methods: UV fluorescence deletion (365 nm/254 nm) and/or by treating the plates with p-anisaldehyde in EtOH, phosphomolybdic acid or permanganate solution, followed by heating. Melting points were obtained on an electrothermal melting point Gallenkamph apparatus and are uncorrected.

NMR spectra were recorded using Agilent MR 400 MHz (1H, 399.85 MHz; 13C, 100.55 MHz) and Agilent VNMRS 500 MHz with cold probe (1H, 400.123 MHz; 13C, 100.611 MHz) spectrometers, at room temperature. High-resolution mass spectra (HRMS) were measured at mass spectrometry service of the Laboratory of Instrumental Techniques, University of Valladolid, on a UPLC-MS system (UPLC: Waters ACQUITY H-class UPLC; MS: Bruker Maxis Impact) by electrospray ionization (ESI positive and negative).

### 3.2. Preparation of Key Compounds

#### 3.2.1. Epoxidation of Vinylsilyl Alcohols **2**-**3** with MCPBA

To a solution of vinylsilyl alcohols **2** or **3** (1 mmol) in dichloromethane, at 0 °C, is added MCPBA (77%, 4 mmol, 896 mg). The mixture is stirred for 1 to 3 h, until the starting materials are consumed (as seen by thin-layer chromatography). The crude is diluted with 15 mL of dichloromethane, washed once with saturated NaHCO_3_ and once with brine. Then, it is dried with anhydrous MgSO_4_ and the solvent is evaporated under reduced pressure. Purification with column chromatography (eluent hexane-ethyl acetate 8 to 1) affords compounds **4-6**.

**Compound 5a:**(1S*)-(dimethylphenylsilyl)((2S*, 5R*)-5-methyltetrahydrofuran-2-yl)methanol. Obtained as a colorless oil in 30.5% chemical yield (152 mg from 1 mmol of **2a**). ^1^H-NMR (CDCl_3_, 400 MHz) δ 7.61–7.54 (m, 2H), 7.37–7.34 (m, 3H), 4.11–4.06 (m, 1H), 4.01–3.97 (m, 1H), 3.36–3.33 (m, 1H), 2.26 (brs, 1H), 1.97–1.81 (m, 1H), 1.75–1.63 (m, 1H), 1.62–1.51 (m, 1H), 1.40–1.34 (m, 1H), 1.18 (d, *J* = 6.1 Hz, 3H), 0.39 (s, 3H), 0.38 (s, 3H); ^13^C-NMR (CDCl_3_, 101 MHz) δ 137.1 (C), 134.3 (2xCH), 129.3 (CH), 127.9 (2xCH), 80.5 (CH), 74.2 (CH), 68.3 (CH), 34.5 CH_2_), 28.2 (CH_2_), 21.0 (CH_3_), −4.4 (CH_3_), −4.8 (CH_3_); HRMS (ESI+) *m/z* calcd. for C_14_H_22_NaO_2_Si ([M + Na]^+^): 273.1281, found 273.1281.

**Compound 6a:**(1S*)-(dimethylphenylsilyl)((2S*,5S*)-5-methyltetrahydrofuran-2-yl)methanol. Obtained as a colorless oil in 30.5% chemical yield (152 mg from 1 mmol of **2a**). ^1^H-NMR (CDCl_3_, 400 MHz) δ 7.61–7.54 (m, 2H), 7.37–7.34 (m, 3H), 3.96–3.92 (m, 2H), 3.35 (d, *J* = 6.8 Hz, 1H), 2.07 (brs, 1H), 1.97–1.81 (m, 1H), 1.75–1.63 (m, 1H), 1.62–1.51 (m, 1H), 1.40–1.34 (m, 1H), 1.19 (d, *J* = 6.1 Hz, 3H), 0.39 (s, 3H), 0.38 (s, 3H); ^13^C-NMR (CDCl_3_, 101 MHz) δ 137.1 (C), 134.3 (2xCH), 129.3 (CH), 127.9 (2xCH), 81.0 (CH), 75.3 (CH), 68.8 (CH), 33.7 CH_2_), 29.0 (CH_2_), 21.5 (CH_3_), −4.4 (CH_3_), −4.8 (CH_3_); HRMS (ESI+) *m*/*z* calcd. for C_14_H_22_NaO_2_Si ([M + Na]^+^): 273.1281, found 273.1289.

**Compound 5b:**(1S*)-(dimethylphenylsilyl)((2S*,5R*)-3,3,5-trimethyltetrahydrofuran-2-yl)methanol. Obtained as a colorless viscous oil in 60% chemical yield (167 mg from 1 mmol of **2b**). ^1^H-NMR (CDCl_3_, 400 MHz) δ 7.63–7.57 (m, 2H), 7.38–7.33 (m, 3H), 4.13–4.05 (m, 1H), 3.53–3.50 (m, 2H), 1.68 (dd, *J* = 11.9, 5.8 Hz, 1H), 1.44 (dd, *J* = 11.9, 9.7 Hz, 1H), 1.18 (d, *J* = 6.0 Hz, 3H), 1.13 (s, 3H), 1.10 (s, 1H, OH),0.99 (s, 3H), 0.38 (s, 3H), 0.37 (s, 3H); ^13^C-NMR (CDCl_3_, 101 MHz) δ 137.3 (C), 134.2 (2xCH), 129.1 (CH), 127.7 (2xCH), 88.3 (CH), 73.3 (CH), 65.0 (CH), 49.3 (CH_2_), 42.3 (C), 29.2 (CH_3_), 24.3 (CH_3_), 21.1 (CH_3_), −4.5 (CH_3_), −4.8 (CH_3_); HRMS (ESI+) *m*/*z* calcd. for C_16_H_26_NaO_2_Si ([M + Na]^+^): 301.1594, found 301.1602.

**Compound 5c:**(1S*)-(dimethylphenylsilyl)((2S*,3S*,5R*)-5-methyl-3-phenyl-tetrahydro-furan-2-yl)methanol. Obtained as a colorless oil in 59% chemical yield (192 mg from 1 mmol of **2c**). ^1^H-NMR (CDCl_3_, 400 MHz) δ 7.60–7.53 (m, 2H), 7.46–7.29 (m, 5H), 7.23–7.17 (m, 3H), 4.34–4.28 (m, 1H), 3.91 (dd, *J* = 8.0 y 2.2 Hz, 1H), 3.50 (q, *J* = 8.0 Hz, 1H), 3.34 (d, *J* = 2.2 Hz, 1H), 2.22–2.17 (m, 1H), 1.98–1.91 (m, 1H), 1.70 (brs, 1H), 1.27 (d, *J* = 6.1 Hz, 3H), 0.37 (s, 3H), 033 (s, 3H); ^13^C-NMR (CDCl_3_, 101 MHz) δ 142.5 (C), 137.2 (C), 134.4 (2xCH), 129.3 (CH), 128.7 (2xCH), 128.0 (2xCH), 127.8 (2xCH), 126.6 (CH), 88.4 (CH), 74.9 (CH), 64.0 (CH), 44.8 (CH), 42.1 (CH_2_), 22.1 (CH_3_), −4.3 (CH_3_), −4.7 (CH_3_); HRMS (ESI+) *m*/*z* calcd. for C_20_H_26_NaO_2_Si ([M + Na]^+^): 349.1594, found 349.1597.

**Compound 4d:**(1S*, 3S*, 4S*, 5S*)-4,5-oxirane-5-dimethylphenylsilyl-1,3,4-triphenyl- -pentan-1-ol. Obtained as a colorless viscous oil in 69% chemical yield (320 mg from 1 mmol of **2d**). Colorless viscous oil. Major isomer:^1^H NMR (CDCl_3,_ 400 MHz) δ 7.51–6.74 (m, 20H), 4.61 (t, *J* = 7.2 Hz, 1H), 2.75 (dd, *J* = 9.0, 6.5 Hz, 1H), 2.51 (s, 1H), 2.47–2.40 (m, 1H), 2.33–2.29 (m, 1H), 1.54 (s, 1H, OH), 0.28 (s, 3H), 0.27 (s, 3H); ^13^C NMR (CDCl_3,_ 101 MHz) δ 144.0 (C), 139.8 (C), 139.3 (C), 136.7 (C), 133.9 (2xCH), 129.6 (CH), 129.1 (2xCH), 128.5 (2xCH), 128.3 (2xCH), 128.0 (2xCH), 127.8 (2xCH), 127.6 (CH),127.4 (CH),127.2 (2xCH), 126.7 (CH),126.1 (2xCH), 72.2 (CH), 69.4 (C), 61.6 (CH), 46.2 (CH), 40.3 (CH_2_), −2.7 (CH_3_), −3.8 (CH_3_).Minor isomer:^1^H NMR (CDCl_3,_ 400 MHz) δ 7.51–6.74 (m, 20H), 4.38 (dd, *J* = 9.5, 4.4 Hz, 1H), 2.52 (s, 1H), 2.47–2.40 (m, 1H), 2.33–2.29 (m, 1H), 2.20–2.12 (m, 1H), 1.54 (s, 1H, OH), 0.19 (s, 3H), 0.12 (s, 3H); ^13^C NMR (CDCl_3,_ 101 MHz) δ 143.7 (C), 138.5 (C), 139.3 (C), 136.8 (C), 133.8 (CH), 129.5 (CH), 129.3 (CH), 128.7 (CH), 128.6 (CH), 128.0 (CH), 127.6 (CH), 127.4 (CH), 127.0 (CH), 126.9 (CH), 126.7 (CH), 126.3 (CH), 72.5 (CH), 69.9 (C), 60.0 (CH), 46.9 (CH), 40.7 (CH_2_), −3.0 (CH_3_), −3.8 (CH_3_); HRMS (ESI+) *m*/*z* calcd. for C_31_H_32_NaO_2_Si ([M + Na]^+^): 487.2064, found 487.2066.

**Compound 4e:**(1R*, 3S*, 4S*, 5S*)-4,5-oxirane-5-dimethylphenylsilyl-1,3,4-triphenyl- pentan-1-ol. Obtained as a white solid (melting point 82.3–84.5 °C) in 65% chemical yield (302 mg from 1 mmol of **3d**). ^1^H NMR (CDCl_3,_ 400 MHz) δ 7.7–6.70 (m, 20H), 4.33–4.30 (m, 1H), 3.15 (dd, *J* = 9.8, 4.8 Hz, 1H), 2.60 (s, 1H), 2.48–2.40 (m, 1H), 2.08–2.01 (m, 1H), 1.25 (s, 1H, OH), 0.57 (s, 6H); ^13^C NMR (CDCl_3,_ 101 MHz) δ 145.0 (C), 139.8 (C), 139.4 (C), 136.7 (C), 134.2 (2xCH), 129.7 (CH), 129.3 (2xCH), 128.7 (2xCH), 128.3 (2xCH), 128.1 (2xCH), 127.8 (2xCH), 127.4 (CH), 127.3 (CH), 127.0 (2xCH), 126.7 (CH), 125.7 (2xCH), 71.9 (CH), 70.3 (C), 61.9 (CH), 46.9 (CH), 40.7 (CH_2_), −2.7 (CH_3_), −2.9 (CH_3_); HRMS (ESI+) *m*/*z* calcd. for C_31_H_32_NaO_2_Si ([M + Na]^+^): 487.2064, found 487.2055.

**Compound 4f:**(4S*, 5S*, 6S*)-5,6-oxirane-6-dimethylphenysilyl-5-phenyl-4-isopropyl- hexan-2-ol. Obtained as a colorless oil in 64% chemical yield (235 mg from 1 mmol of **3e**). ^1^H NMR (CDCl_3,_ 400 MHz) δ 7.70–7.12 (m, 10H), 4.36–4.26 (m, 1H), 2.53 (s, 1H), 2.37 (brs, 1H, OH), 1.84–1.76 (m, 1H), 1.47–1.27 (m, 3H), 1.14 (d, *J* = 6.3 Hz, 3H), 0.97 (d, *J* = 6.8 Hz, 3H), 0.53 (s, 6H), 0.48 (d, *J* = 6.8 Hz, 3H); ^13^C NMR (CDCl_3,_ 101 MHz) δ 140.2 (C), 136.3 (C), 134.0 (2xCH), 129.8 (CH), 128.1 (2xCH), 128.0 (2xCH), 127.8 (2xCH), 127.5 (CH), 69.5 (C), 65.0 (CH), 59.9 (CH), 45.3 (CH), 37.2 (CH_2_), 29.6 (CH), 23.2 (CH_3_), 23.0 (CH_3_), 18.8 (CH_3_), −2.8 (CH_3_), −2.9 (CH_3_); HRMS (ESI+) *m*/*z* calcd. for C_23_H_32_NaO_2_Si ([M + Na]^+^): 391.2064, found 391.2062.

**Compound 4g:***(3R, 4S, 5S)-4,5-oxirane-5-dimethylphenysilyl-4-phenyl-3-methyl- hexan-2-ol.* Obtained as a yellow oil in 62% chemical yield (202 mg from 1 mmol of **2b**). Yellow oil. ^1^H NMR (CDCl_3_, 500 MHz) δ 7.65–7.60 (m, 2H), 7.44–7.39 (m, 3H), 7.35–7.23 (m, 5H), 3.75–3.70 (m, 1H), 3.54–3.48 (m, 1H), 2.50 (s, 1H), 1.81 (brs, 1H), 1.68–1.56 (m, 2H), 1.30–1.222 (m, 1H), 0.90 (d, *J* = 6.9 Hz, 3H), 0.53 (s, 3H), 0.52 (s, 3H); ^13^C NMR (CDCl_3_, 101 MHz) δ 139.1 (C), 136.5 (C), 133.9 (2xCH), 129.7 (CH), 128.1 (2xCH), 127.9 (2xCH), 127.5 (2xCH), 127.4 (CH), 69.7 (C), 61.1 (CH_2_), 59.5 (CH), 36.1 (CH_2_), 35.9 (CH), 18.7 (CH_3_), −3.0 (CH_3_), −3.1 (CH_3_); HRMS (ESI+) *m*/*z* calcd. for C_20_H_26_NaO_2_Si ([M + Na]^+^): 349.1594, found 349.1598.

#### 3.2.2. Acid-Catalyzed Intramolecular Cyclization of Epoxysilyl Alcohol **4e**

To a solution of the epoxysilyl alcohol **4e** (0.2 mmol) in dichloromethane is added the corresponding acid (BF_3_.OEt_2_, pTSA, CSA, Table 2). The reaction is stirred until complete conversion of the starting material is observed by TLC. Then, it is hydrolyzed with saturated NaHCO_3_. Layers are separated, and the aqueous layer is extracted three times with dichloromethane, dried over anhydrous MgSO_4_ and the solvent is evaporated under reduced pressure.Purification with column chromatography (eluent hexane-ethyl acetate 8 to 1) affords compounds **7**-**10**.

**Compound 7:**(3S*,4R*,6R*)-3,4,6-triphenyltetrahydro-2H-pyran-2-ol. Obtained as a colorless oil in 72% chemical yield (47 mg from 0.2 mmol of **4e** and BF_3_.OEt_2_, entry 5, Table 2). *α Isomer*: ^1^H NMR (CDCl_3,_ 400 MHz) δ 7.31–6.95 (m, 15H), 5.54 (d, *J* = 3.1 Hz, 1H), 3.75 (dd, *J* = 11.3, 3.1 Hz, 1H), 3.42 (td, *J* = 11.7, 4.1 Hz, 1H), 3.35–3.25 (m, 1H), 1.76–1.58 (m, 2H), 1.25 (s, 1 H, OH); ^13^C NMR (CDCl_3,_ 101 MHz) δ 143.5 (C), 142.5 (C), 142.2 (C), 141.9 (C), 139.5 (C), 138.8 (C), 129.9 (2xCH), 128.9 (CH), 128.8 (CH),128.2 (4xCH), 128.1 (2xCH), 128.0 (2xCH), 127.9 (2xCH), 127.6 (2xCH), 127.5 (2xCH), 127.4 (2xCH),127.2 (CH), 126.9 (CH), 126.5 (CH), 126.2 (CH), 126.0 (2xCH), 126.0 (CH), 125.9 (CH), 125.1 (2xCH), 96.3 (CH), 70.7 (CH), 51.5 (CH), 43.6 (CH_2_), 40.2 (CH). *β Isomer*: ^1^H NMR (CDCl_3,_ 400 MHz) δ 7.31–6.95 (m, 15H), 5.06 (d, *J* = 8.4 Hz, 1H), 4.66 (d, *J* = 11.0, 1H), 3.35–3.25 (m, 1H), 3.02 (dd, *J* = 11.8, 8.4 Hz 1H), 2.11–2.03 (m, 1H), 1.76–1.58 (m, 1H), 1.25 (s, 1 H, OH); ^13^C NMR (CDCl_3,_ 101 MHz) δ 143.5 (C), 142.5 (C), 142.2 (C), 141.9 (C), 139.5 (C), 138.8 (C), 129.9 (2xCH), 128.9 (CH), 128.8 (CH),128.2 (4xCH), 128.1 (2xCH), 128.0 (2xCH), 127.9 (2xCH), 127.6 (2xCH), 127.5 (2xCH), 127.4 (2xCH),127.2 (CH), 126.9 (CH), 126.5 (CH), 126.2 (CH), 126.0 (2xCH), 126.0 (CH), 125.9 (CH), 125.1 (2xCH), 125.1 (CH), 99.6 (CH), 75.9 (CH), 53.4 (CH), 51.5 (CH), 42.6 (CH_2_); HRMS (ESI+) *m*/*z* calcd. for C_23_H_22_NaO_2_ ([M + Na]^+^): 353.1512, found 353.1518.

**Compound 8:**(3R*,4R*,6R*)-3,4,6-triphenyltetrahydro-2H-pyran-2-ol. Obtained as a colorless oil in 18% chemical yield (12 mg from 0.2 mmol of **4e** and BF_3_.OEt_2_, entry 6, Table 2). *α Isomer*: ^1^H NMR (CDCl_3_, 400 MHz) δ 7.47–7.03 (m, 15H), 5.53 (d, *J* = 2.7 Hz, 1H), 5.37 (dd, *J* = 11.8, 2.4 Hz, 1H), 3.87 (td, *J* = 12.4, 3.7 Hz, 1H), 3.39 (dd, *J* = 12.4, 2.7 Hz, 1H), 2.47 (s, 1H, OH), 2.21–2.12 (m, 1H), 1.90–0.82 (m, 1H); ^13^C NMR (CDCl_3_, 101 MHz) δ 142.1 (C), 141.0 (C), 138.8 (C), 129.4 (CH), 128.4 (2xCH), 128.3 (2xCH), 128.0 (CH), 127.5 (2xCH), 127.4 (CH),126.7 (CH), 126.5 (CH),126.4 (CH), 126.1 (CH), 126.0 (CH), 125.8 (CH), 95.5 (CH), 71.0 (CH), 51.8 (CH), 43.4 (CH_2_), 39.6 (CH). *β Isomer*: ^1^H NMR (CDCl_3_, 400 MHz) δ 7.47–7.03 (m, 15H), 5.20 (d, *J* = 8.4 Hz, 1H), 4.86 (dd, *J* = 11.1, 2.4 Hz, 1H), 3.30 (td, *J* = 11.9, 3.9 Hz, 1H), 2.92 (dd, *J* = 11.9, 8.4 Hz, 1H), 2.81 (s, 1H, OH), 2.21–2.12 (m, 1H), 2.05–1.94 (m, 1H); ^13^C NMR (CDCl_3_, 101 MHz) δ 142.1 (C), 141.0 (C), 138.8 (C), 129.4 (CH), 128.4 (2xCH), 128.3 (2xCH), 128.0 (CH), 127.5 (2xCH), 127.4 (CH),126.7 (CH), 126.5 (CH),126.4 (CH), 126.1 (CH), 126.0 (CH), 125.8 (CH), 99.9 (CH), 77.7 (CH), 55.2 (CH), 47.5 (CH), 41.8 (CH_2_); HRMS (ESI+) *m*/*z* calcd. for C_23_H_22_NaO_2_ ([M + Na]^+^): 353.1512, found 353.1518.

**Compound 9:**(2R*,4S*)-2,4,5-triphenyl-3,4-dihydro-2H-pyran. Obtained as a white solid, melting point 128–131 °C, in 33% chemical yield (21 mg from 0.2 mmol of **4e** and BF_3_.OEt_2_, entry 5, Table 2). ^1^H NMR (CDCl_3_, 400 MHz) δ 7.41–6.99 (m, 15H), 7.10 (br s, 1H), 5.09 (d, *J* = 11.5 Hz, 1H), 4.27 (dd, *J* = 11.0, 6.6 Hz, 1H), 2.50 (dd, *J* = 13.9, 6.6 Hz, 1H), 2.10 (dt, *J* = 13.9, 11.5 Hz, 1H); ^13^C NMR (CDCl_3,_ 101 MHz) δ 144.8 (CH), 143.6 (C), 140.7 (C), 138.3 (C), 128.5 (2xCH), 128.3 (2xCH), 128.0 (2xCH), 127.9 (CH), 127.8 (2xCH), 126.0 (3xCH), 126.0 (2xCH), 125.6 (CH), 117.0 (C), 77.7 (CH), 42.3 (CH_2_), 41.5 (CH); HRMS (ESI+) *m*/*z* calcd. for C_23_H_20_NaO ([M + Na]^+^): 335.1406, found 335.1410.

**Compound 10:**(2R*,4S*)-6-dimethylphenyl-2,4,5-triphenyl-3,4-dihydro-2H-pyran. Obtained as a colorless oil in 4.5% chemical yield (4 mg from 0.2 mmol of **4e** and BF_3_.OEt_2_, entry 1, Table 2). ^1^H NMR (CDCl_3_, 400 MHz) δ 7.50–6.69 (m, 20H), 5.03 (dd, *J* = 11.6, 1.9 Hz, 1H), 3.91 (dd, *J* = 11.3, 7.0 Hz, 1H), 2.50 (ddd, *J* = 13.7, 7.0, 1.9 Hz, 1H), 2.26 (dt, *J* = 13.7, 11.6 Hz, 1H), 0.01 (s, 3H), −0.01 (s, 3H); ^13^C NMR (CDCl_3_, 101 MHz) δ 143.1 (C), 142.2 (C), 133.7 (2xCH), 130.9 (2xCH), 128.6 (CH), 128.5 (2xCH), 128.2 (2xCH), 128.0 (2xCH), 127.5 (2xCH), 127.3 (CH), 127.1 (2xCH), 126.3 (CH), 126.1 (CH), 125.6 (2xCH), 76.7 (CH), 46.7 (CH), 41.3 (CH_2_), −2.3 (Si-CH_3_), −2.7 (Si-CH_3_); HRMS (ESI+) *m*/*z* calcd. for C_31_H_30_NaOSi ([M + Na]^+^): 469.1958, found 469.1966.

## 4. Conclusions

In conclusion, a study on different factors that may influence the outcome of the acid-catalyzed cyclization of silylepoxy alcohols is reported. Interestingly, the reaction with MCPBA of *cis*-1,2-disubstituted vinylsilyl alcohols provides tetrahydrofurans through a tandem epoxidation-cyclization reaction, while trisubstituted vinylsilyl alcohols afford the expected epoxysilyl alcohols. Surprisingly, the acid catalyzed cyclization of these hindered epoxysilyl alcohols gives tetrahydropyran derivatives, which are the products of a side isomerization-cyclization reaction. A mechanistic proposal for the formation of all the products is proposed.

## Figures and Tables

**Table 1:**
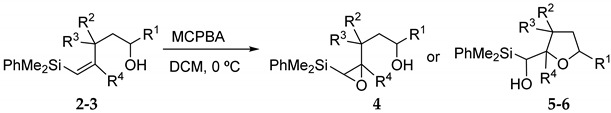
Reaction of vinylsilyl alcohols **2-3** with MCPBA.

Entry	Vinylsilyl Alcohol	Product	Yield (%)
1	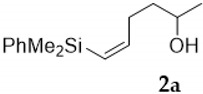	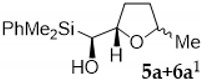	60
2	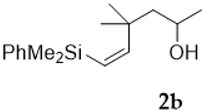	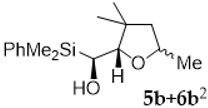	61
3	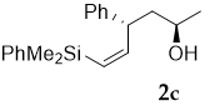	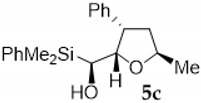	59
4	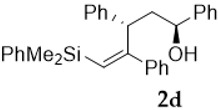	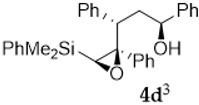	69
5	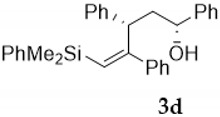	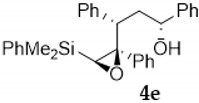	65
6	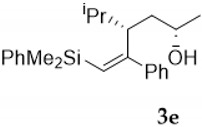	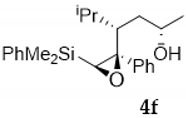	64
7	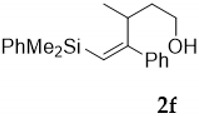	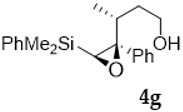	62

^1^ **5a+6a** were obtained as a 50:50 mixture of epimers in C-5. ^2^ **5b+6b** were obtained as a 80:20 mixture of epimers in C-5. ^3^ **4d** was obtained together with the corresponding epoxidation product from the bottom face in a ratio of 90:10.

**Table 2:**
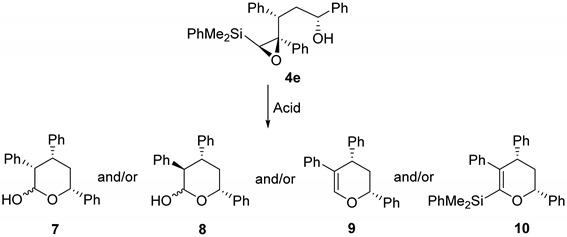
Acid catalyzed cyclization of epoxysilyl alcohol **4e**.

Entry	Acid	Solvent	Conditions	Product Ratio 7/8/9/10	Yield (%)
1	pTSA	CH_2_Cl_2_	0 °C, 2 h	0/15/80/5	88
2	pTSA	CH_2_Cl_2_	−40 °C, 4 h	-----	n.r. ^1^
3	CSA	CH_2_Cl_2_	0 °C, 4 h	0/27/73/0	78
4	H_2_O	H_2_O	r.t., 3 days	0/29/71/0	46
5	BF_3_.OEt_2_	CH_2_Cl_2_	0 °C, 30 min	60/0/40/0	83
6	BF_3_.OEt_2_	CH_2_Cl_2_	−40 °C, 30 min	60/40/0/0	40
7	BF_3_.OEt_2_	CH_2_Cl_2_	−78 °C, 30 min	30/70/0/0	35

^1^ n.r. stands for no reaction.

## Data Availability

The data presented in this study are available in Supplementary Material.

## Supplementary Materials

Data for X-ray structure of **4e** and **9** and copies of NMR spectra of new compounds are available online.

## Sample Availability

Samples of the compounds are not available from the authors.
