# Supplementary material for: Influence of the Substituents on the Opening of Silylepoxy Alcohols: 5-exo-Cyclization towards Tetrahydrofurans vs. Unexpected Side Reaction Leading to Tetrahydropyrans
Source: Molecules. 2021 Dec 6;26(23):7386. doi: 10.3390/molecules26237386 (PMC8658919; doi:10.3390/molecules26237386)

SUPPORTING INFORMATION

Influence of the substituents on the opening of silylepoxy alcohols: 5-*exo* cyclization towards tetrahydrofurans *vs* unexpected side reaction leading to tetrahydropyrans

Carlos Díez-Poza and Asunción Barbero*

Department of Organic Chemistry, Faculty of Science, University of Valladolid, Campus Miguel Delibes, 47011, Valladolid, SPAIN.

E-mail: asuncion.barbero@uva.es

Web: http://organosilanesorganicsynthesis.blogs.uva.es/

Table of Contents

1. X-Ray Crystallographic Data of compound **4e** and **8** S-2

2. Copies of NMR Spectra S-6

3.1 X-Ray Crystallographic Data of compound 4e

The crystal structure has been deposited at the Cambridge Crystallographic Date Center and allocated the deposition number CCDC: 2118173. This data can be obtained free of charge from the Cambridge Crystallographic Date Center via www.ccdc.cam.ac.uk/data_request/ci

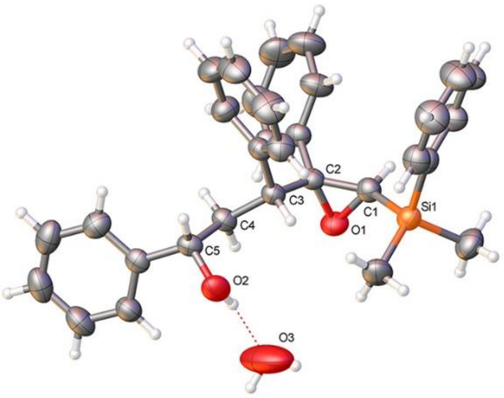

Figure 2. X-ray crystal structure of (1R*, 3S*, 4S*, 5S*)-4,5-epoxy-5-dimethyl(phenyl)silyl-1,3,4-triphenyl-pentan-1-ol **4e**

Experimental

Single crystals of $C_{31}H_{34}O_3Si$ (**4e**) were plates. A suitable crystal was selected and kept at 296.15 K during data collection. Using Olex2, the structure was solved with the ShelXT structure solution program using Direct Methods and refined with the ShelXL refinement package using Least Squares minimization.

X-Ray crystallographic data of **4e** (Figure 2). Crystal data and structure refinement for **4e**

```
Bond precision:  C-C = 0.0029 A         Wavelength=1.54184

Cell:           a=9.31052(11)    b=18.41150(17)     c=16.27509(14)
                alpha=90         beta=97.2884(9)    gamma=90
Temperature:    296 K

                Calculated                 Reported Volume
                2767.35(5)                 2767.35(5)
Space group     P 21/n                     P 1 21/n 1
Hall group      -P 2yn                     -P 2yn
Moiety formula  C31 H32 O2 Si, H2 O        C31 H32 O2 Si, H2 O Sum
formula         C31 H34 O3 Si              C31 H34 O3 Si
Mr              482.67                     482.67
Dx,g cm-3       1.158                      1.159
Z               4                          4
Mu (mm-1)       0.967                      0.967
F000            1032.0                     1032.0
F000'           1035.73
h,k,lmax        11,23,20                   11,23,20
Nref            5686                       5634
Tmin,Tmax       0.612,0.722                0.818,0.888
Tmin'           0.541

Correction method= # Reported T Limits: Tmin=0.818 Tmax=0.888
AbsCorr = GAUSSIAN

Data completeness= 0.991          Theta(max)= 74.811

R(reflections)= 0.0577( 4945)     wR2(reflections)= 0.1696( 5634)

S = 1.040                  Npar= 323
```

3.2 X-Ray Crystallographic Data of compound 9

The crystal structure has been deposited at the Cambridge Crystallographic Date Center and allocated the deposition number CCDC: 2118174. This data can be obtained free of charge from the Cambridge Crystallographic Date Center via www.ccdc.cam.ac.uk/data_request/ci

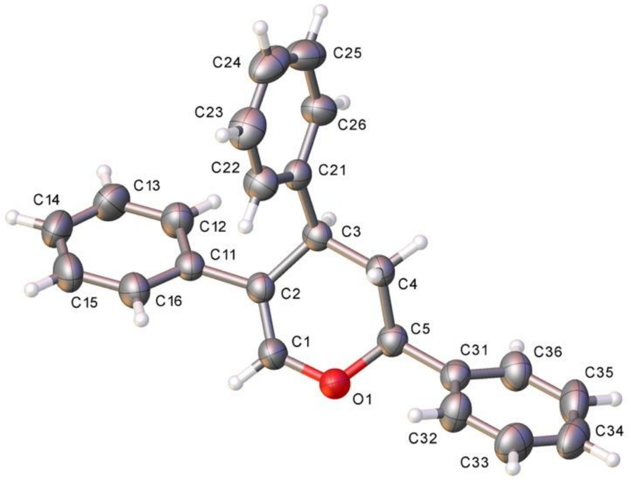

Figure 3. X-ray crystal structure of (2R*,4S*)-2,4,5-triphenyl-3,4-dihydro-2H-pyran **9**

Experimental

Single crystals of $C_{23}H_{20}O$ (**9**) were plates. A suitable crystal was selected and kept at 298.15 K during data collection. Using Olex2, the structure was solved with the ShelXT structure solution program using Direct Methods and refined with the ShelXL refinement package using Least Squares minimisation.

X-Ray crystallographic data of **9** (Figure 3). Crystal data and structure refinement for **9**

```
Bond precision:  C-C = 0.0023 A          Wavelength=0.71073

Cell:            a=16.6186(11)     b=6.2601(4)         c=16.7987(12)
                 alpha=90          beta=100.025(7)     gamma=90
Temperature:     298 K

                    Calculated                 Reported
Volume           1721.0(2)                  1720.9(2)
Space group      P 21/c                     P 1 21/c 1
Hall group       -P 2ybc                    -P 2ybc
Moiety formula   C23 H20 O                  C23 H20 O
Sum formula      C23 H20 O                  C23 H20 O
Mr               312.39                     312.39
Dx,g cm-3        1.206                      1.206
Z                4                          4
Mu (mm-1)        0.072                      0.072
F000             664.0                      664.0
F000'            664.27
h,k,lmax         23,8,23                    22,8,23
Nref             4876                       4026
Tmin,Tmax        0.976,0.985                0.981,0.987
Tmin'            0.975

Correction method= # Reported T Limits: Tmin=0.981 Tmax=0.987 AbsCorr =
GAUSSIAN

Data completeness= 0.826          Theta(max)= 29.698

R(reflections)= 0.0479( 2752)     wR2(reflections)= 0.1192( 4026)

S = 1.023                Npar= 218
```

4. NMR spectra of compounds

^{1}H NMR (400 MHz, CDCl₃)

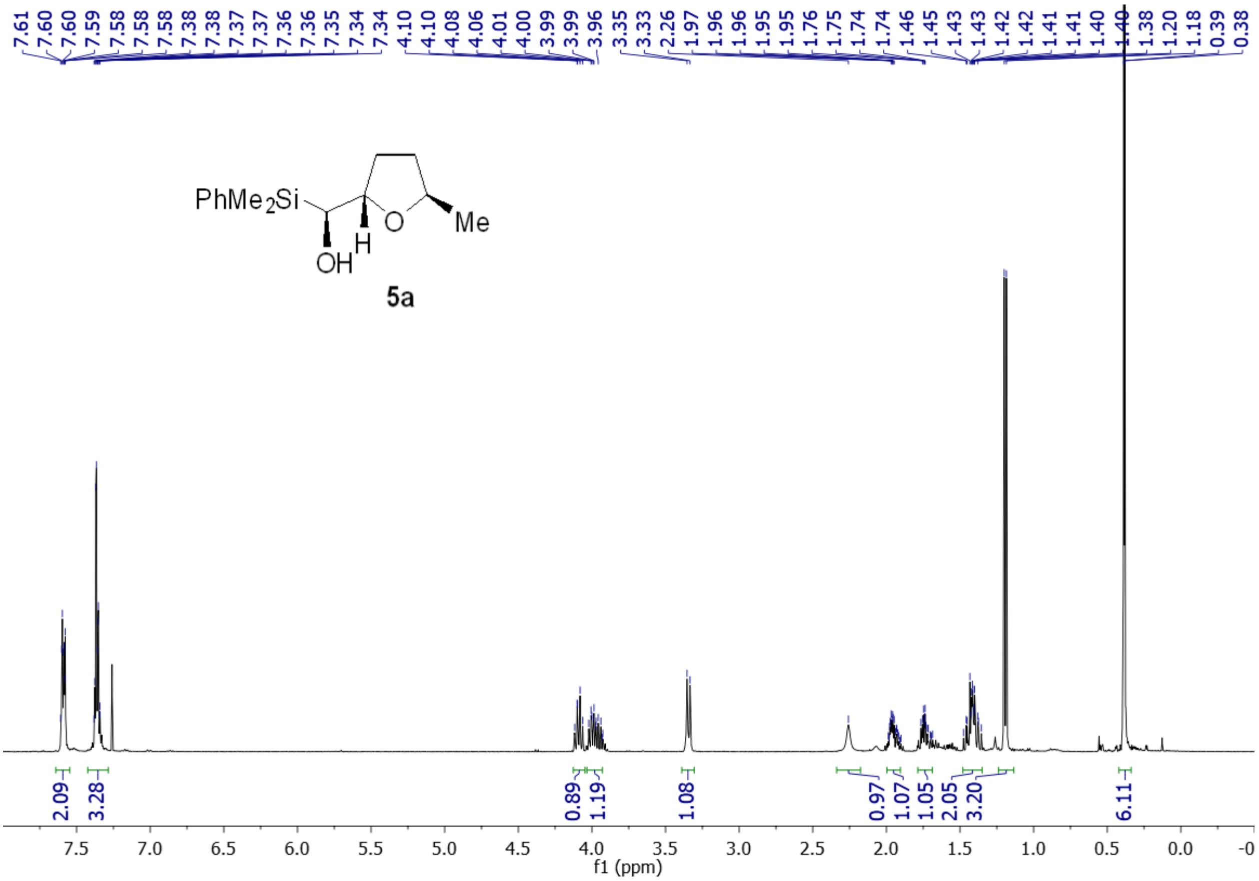

^{13}C NMR (101 MHz, CDCl₃)

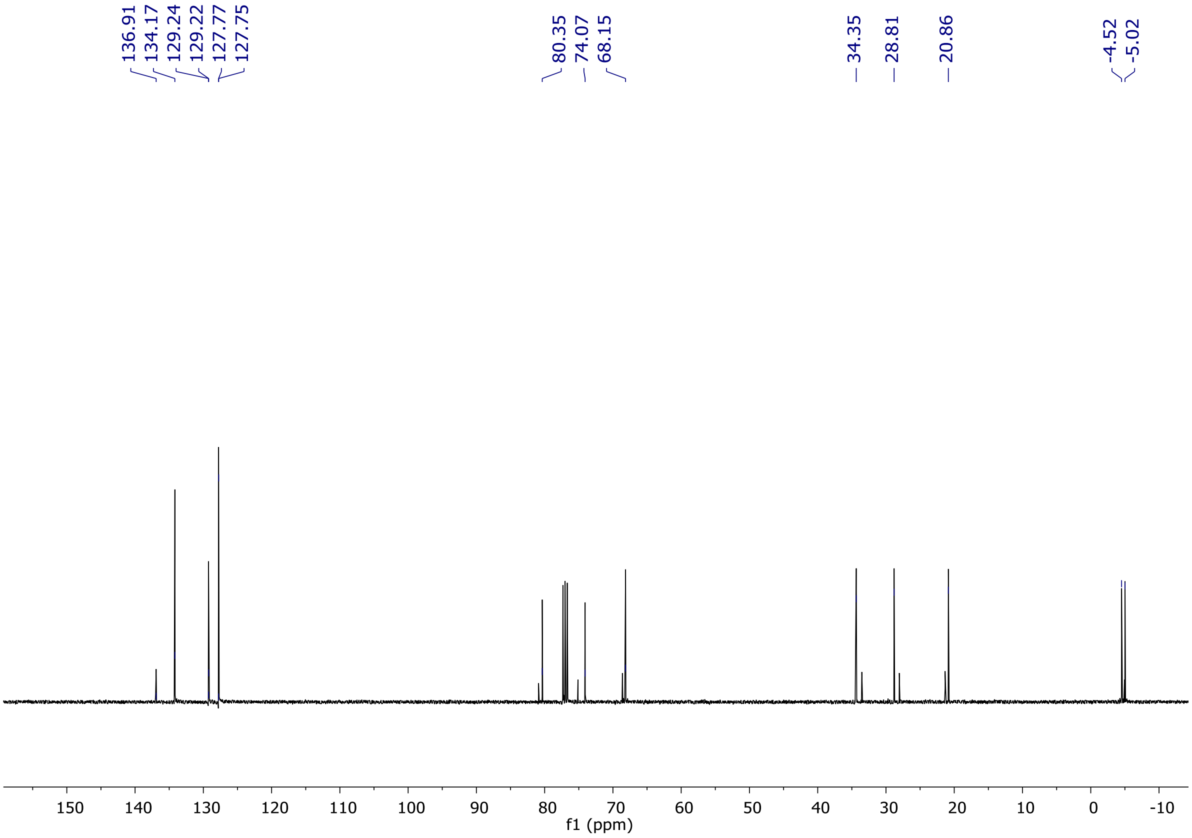

^{1}H NMR (400 MHz, $CDCl_3$)

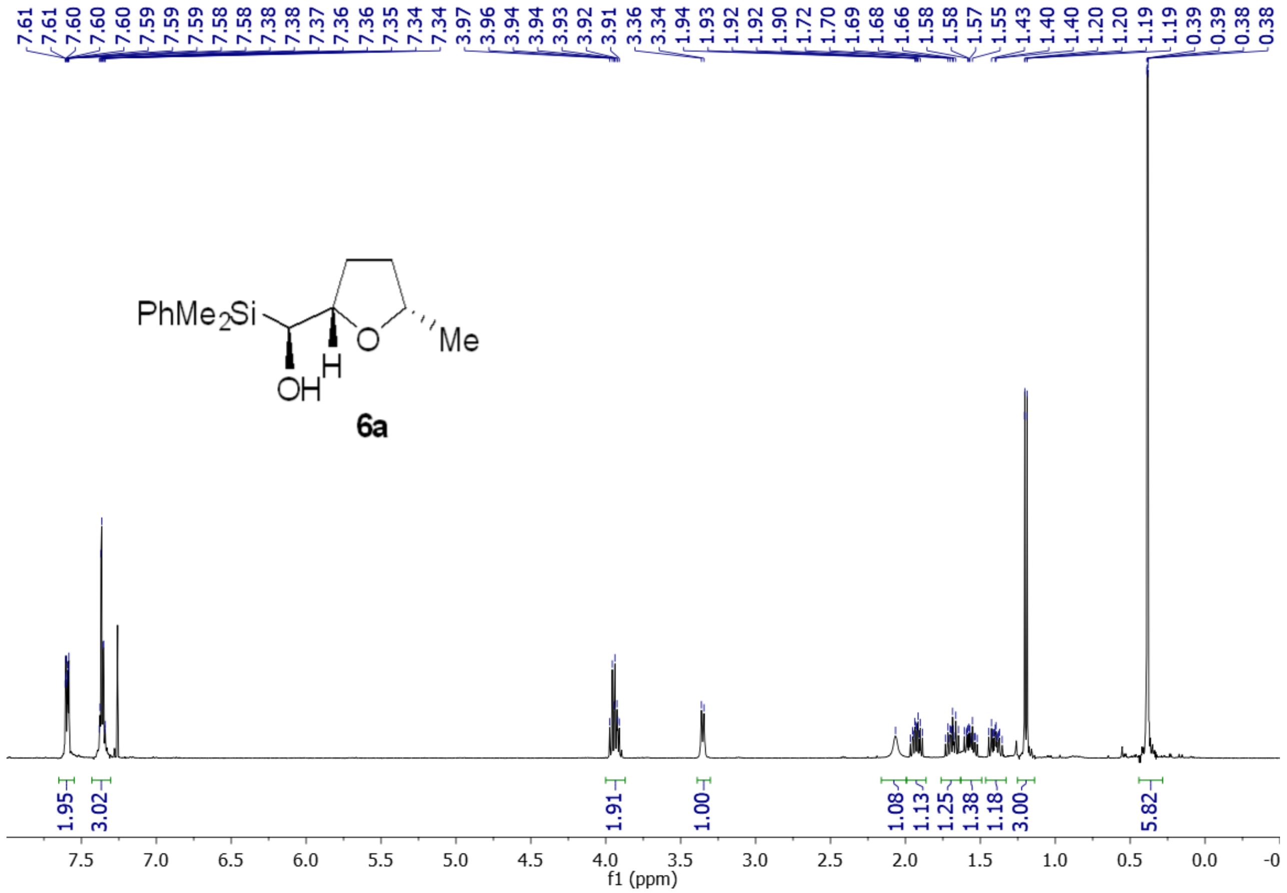

^{13}C NMR (101 MHz, $CDCl_3$)

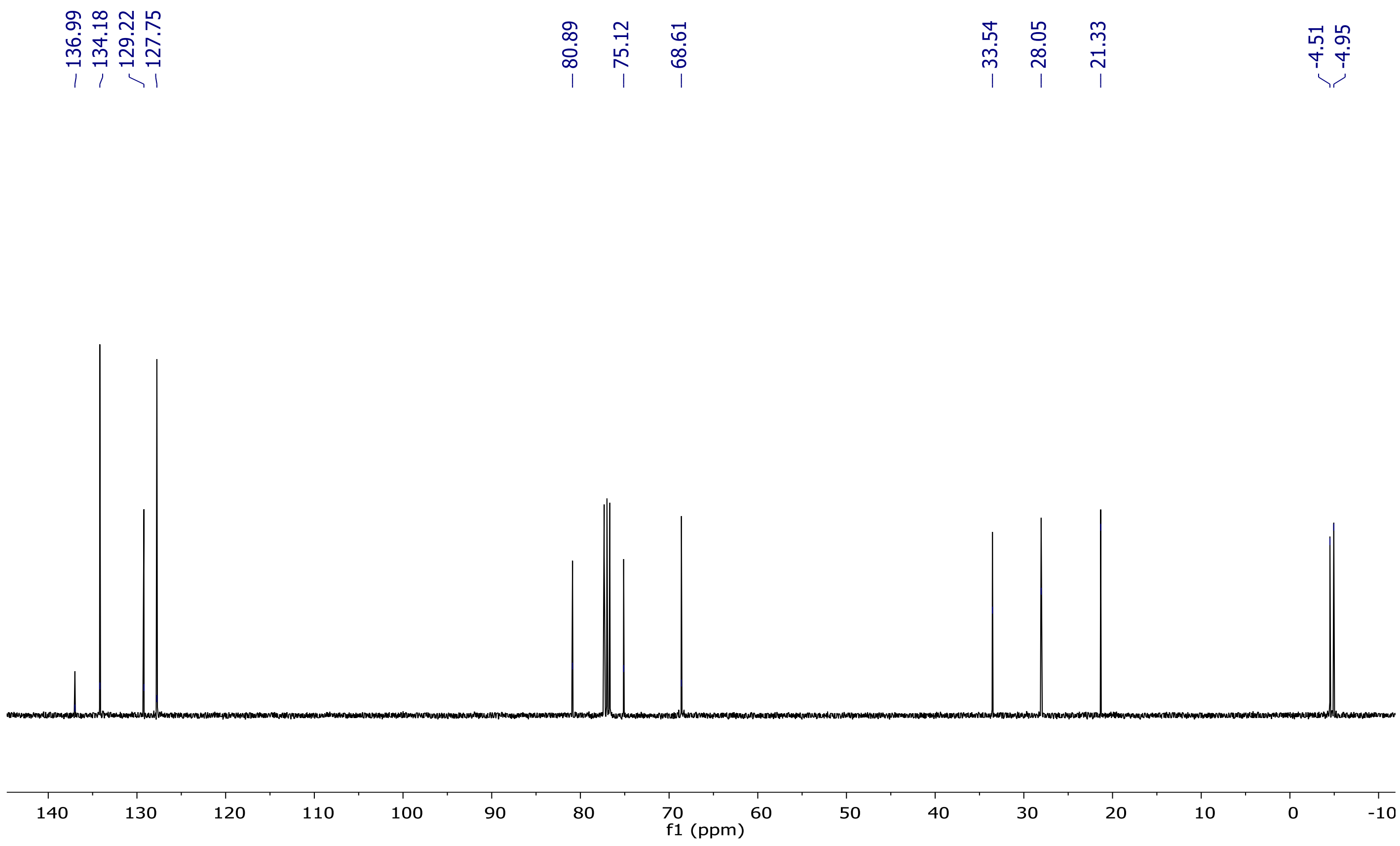

^{1}H NMR (400 MHz, $CDCl_3$)

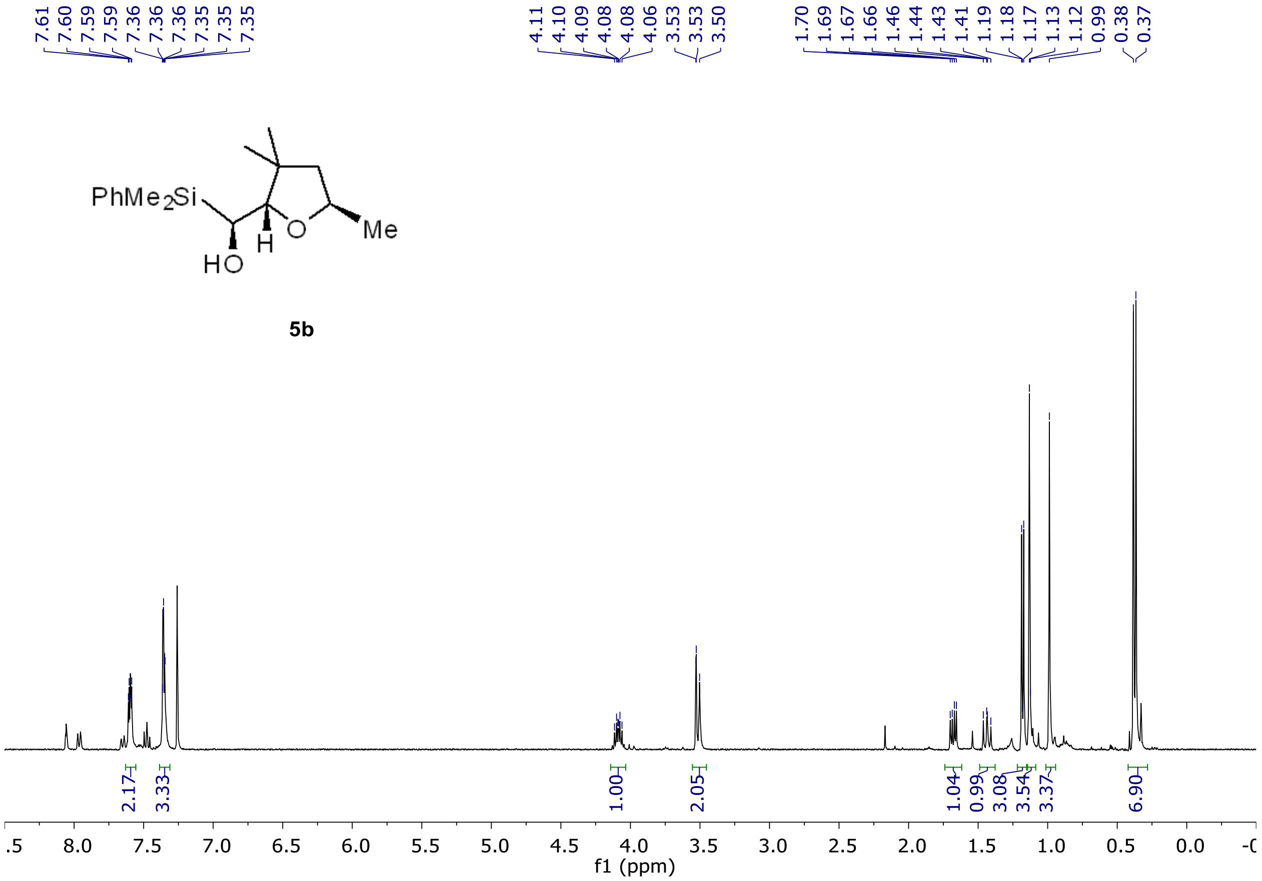

5b

^{13}C NMR (101 MHz, $CDCl_3$)

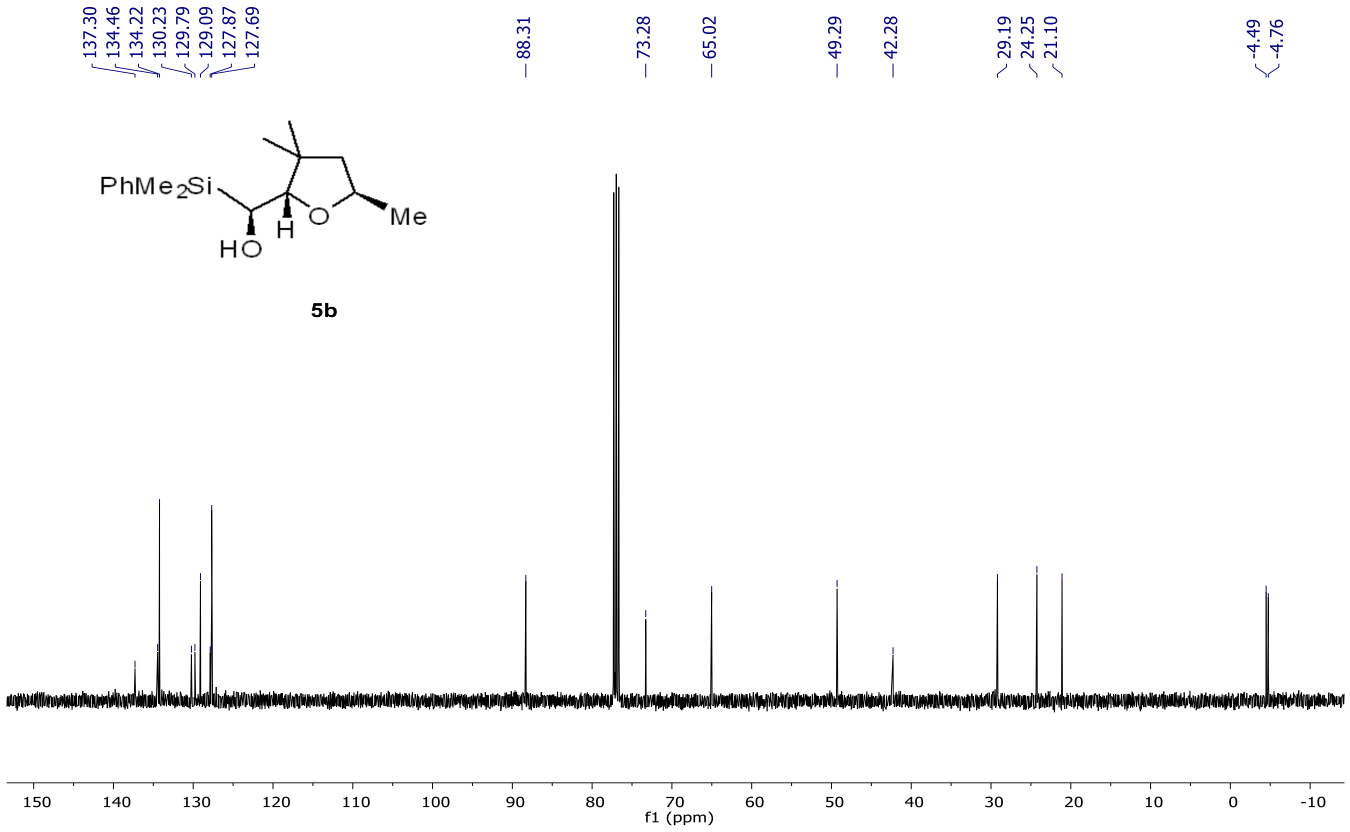

5b

^{1}H NMR (400 MHz, $CDCl_3$)

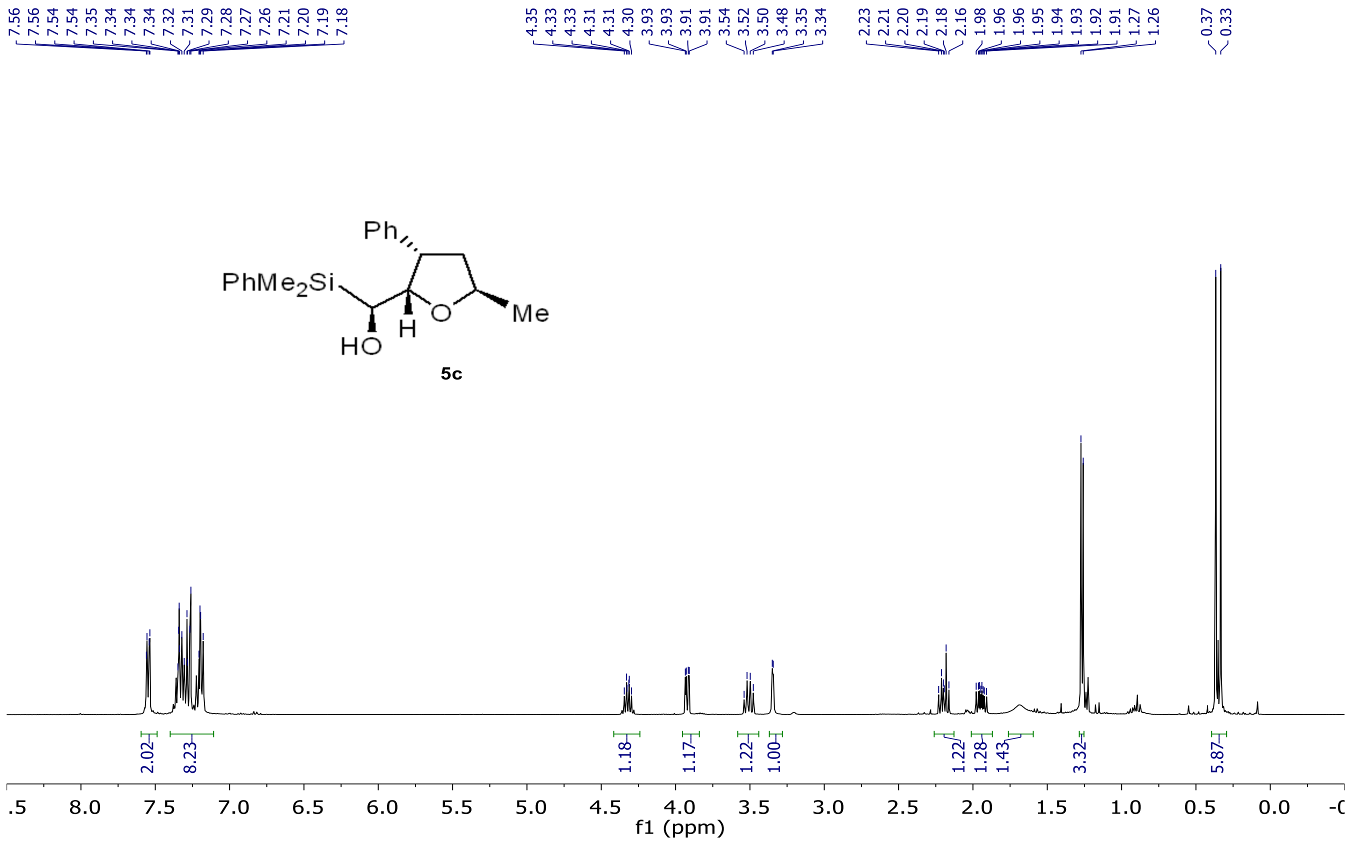

^{13}C NMR (101 MHz, $CDCl_3$)

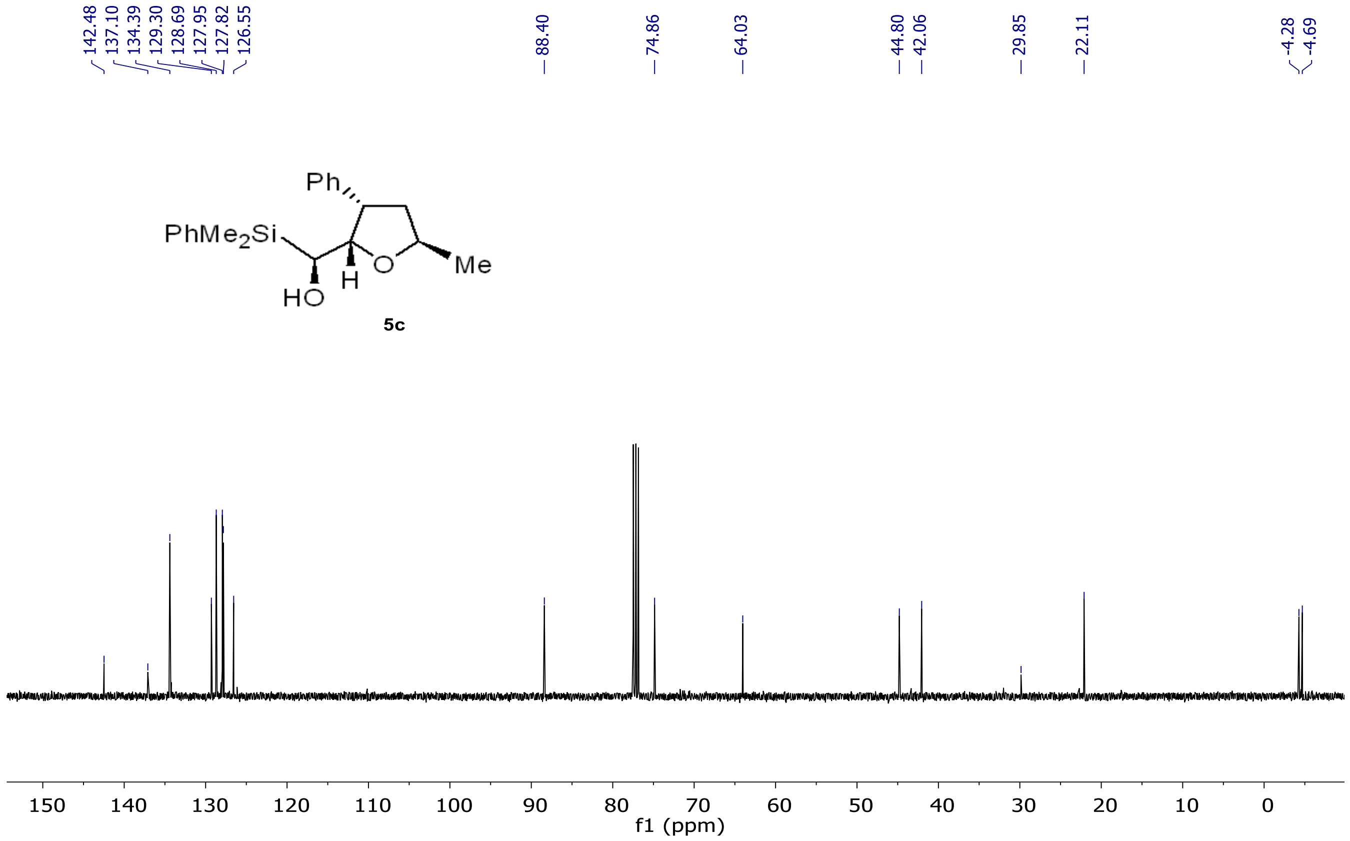

^{1}H NMR (400 MHz, CDCl3)

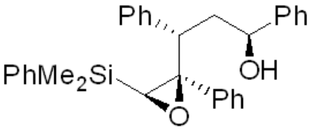

4d (+ minor)

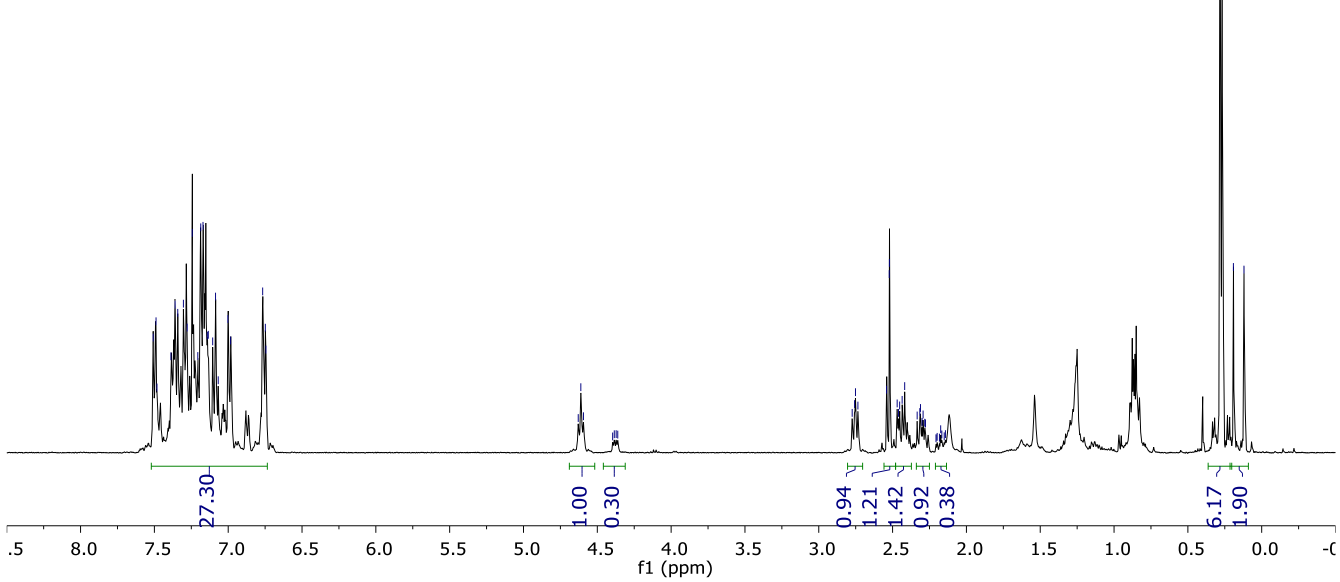

^{13}C NMR (101 MHz, CDCl3)

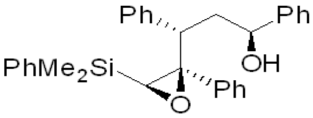

4d (+ minor)

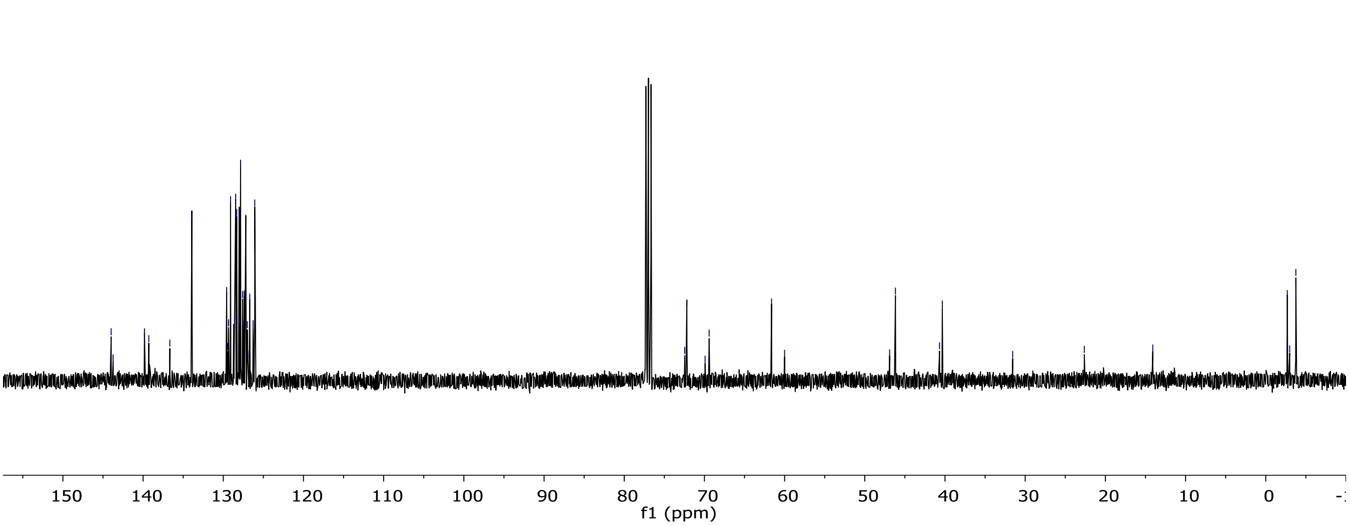

^{1}H NMR (400 MHz, $CDCl_3$)

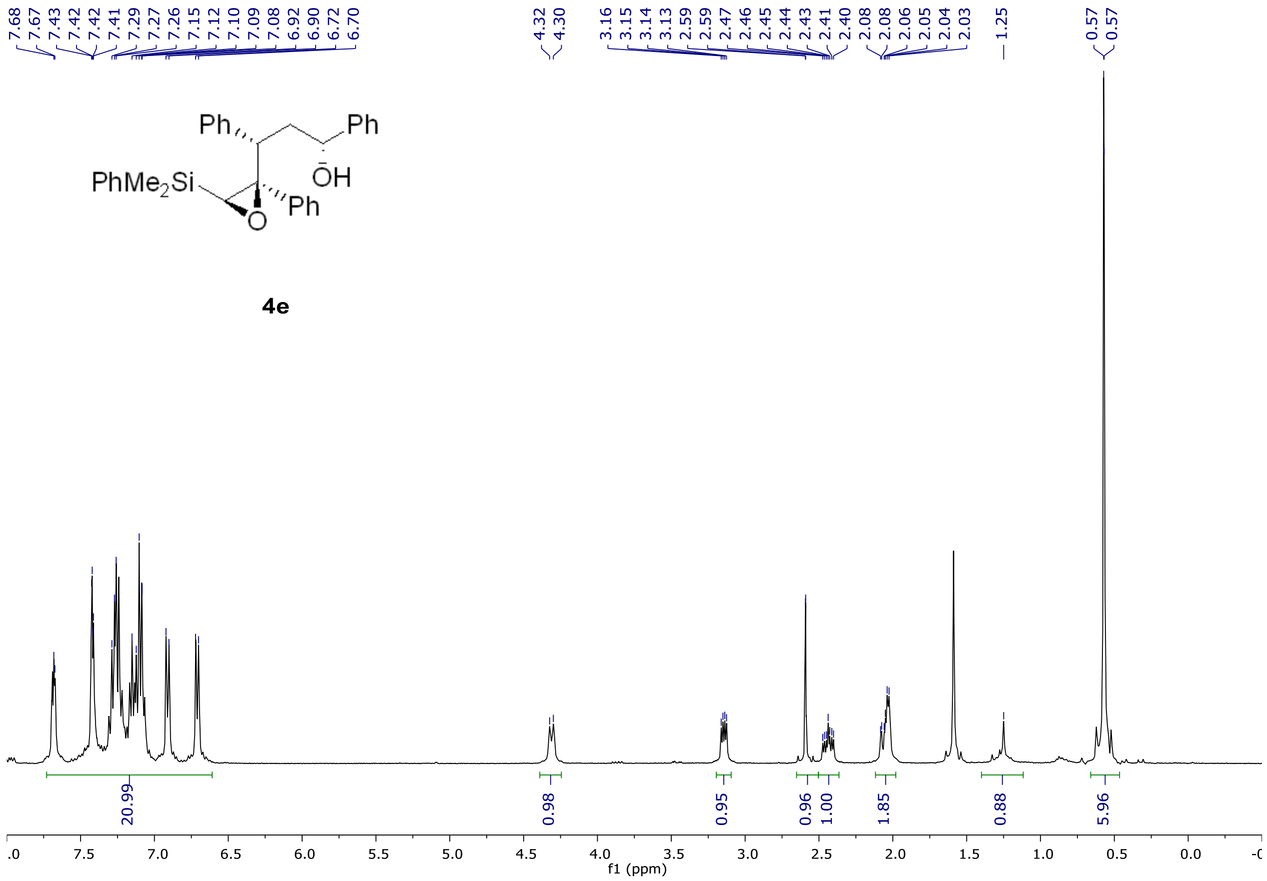

^{13}C NMR (101 MHz, $CDCl_3$)

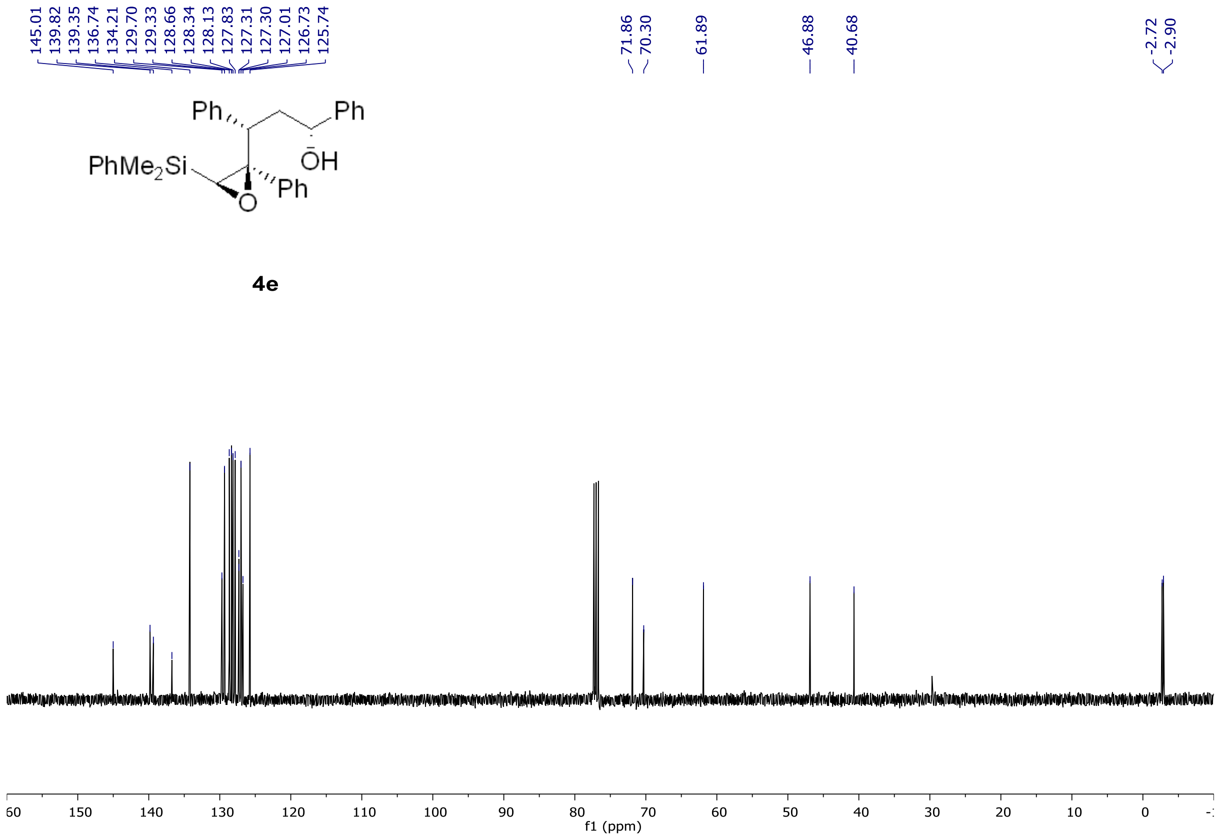

^{1}H NMR (400 MHz, CDCl$_3$)

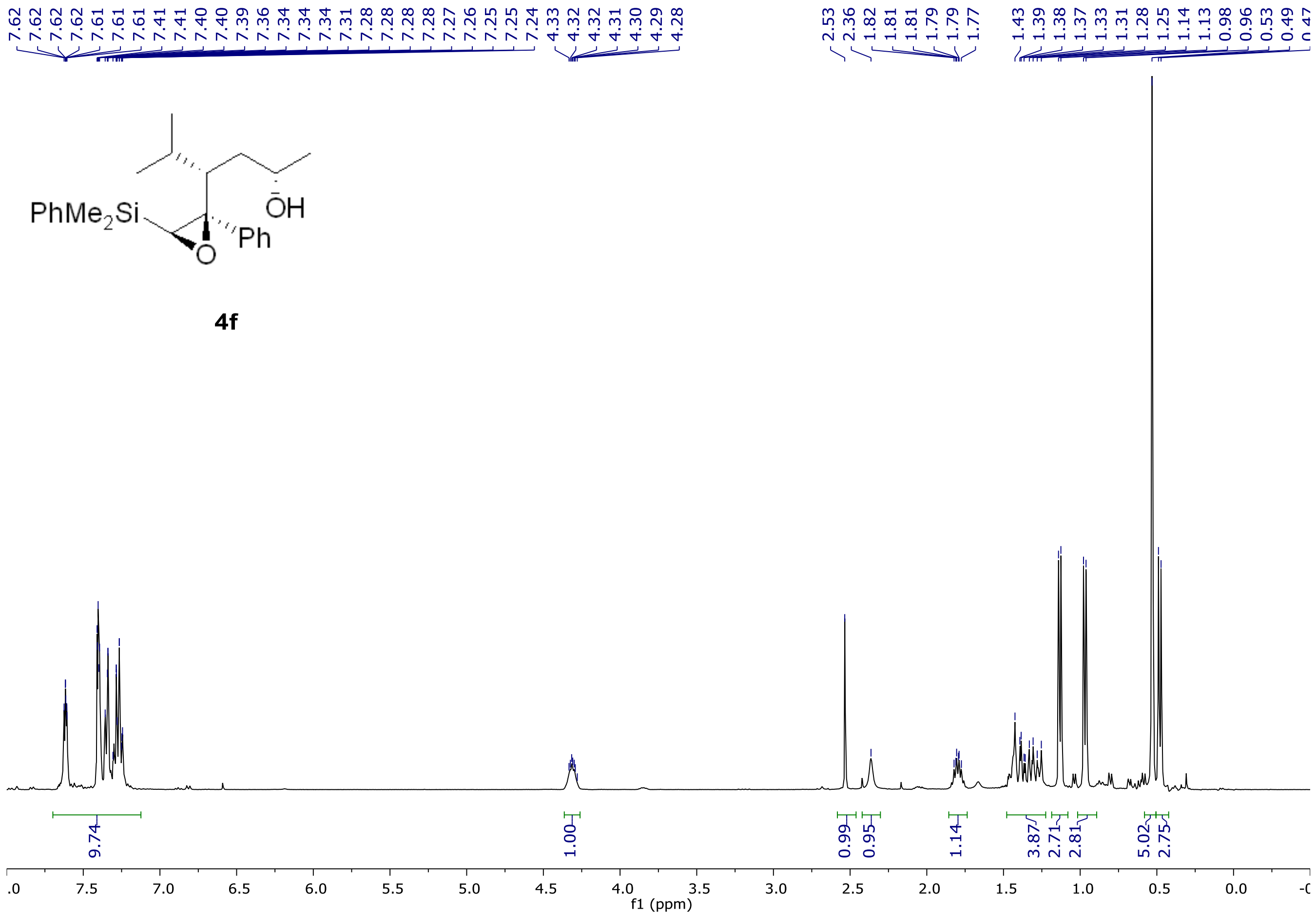

^{13}C NMR (101 MHz, CDCl$_3$)

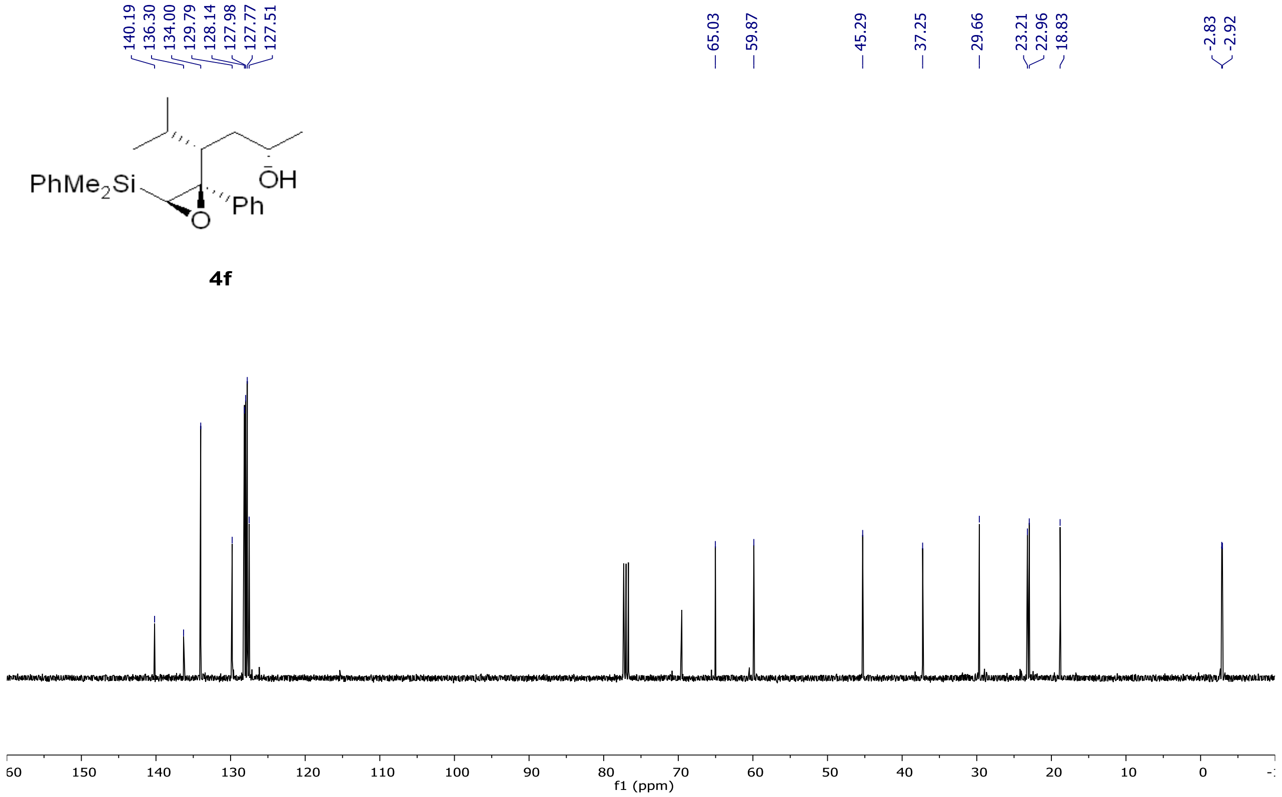

^{1}H NMR (400 MHz, CDCl$_3$)

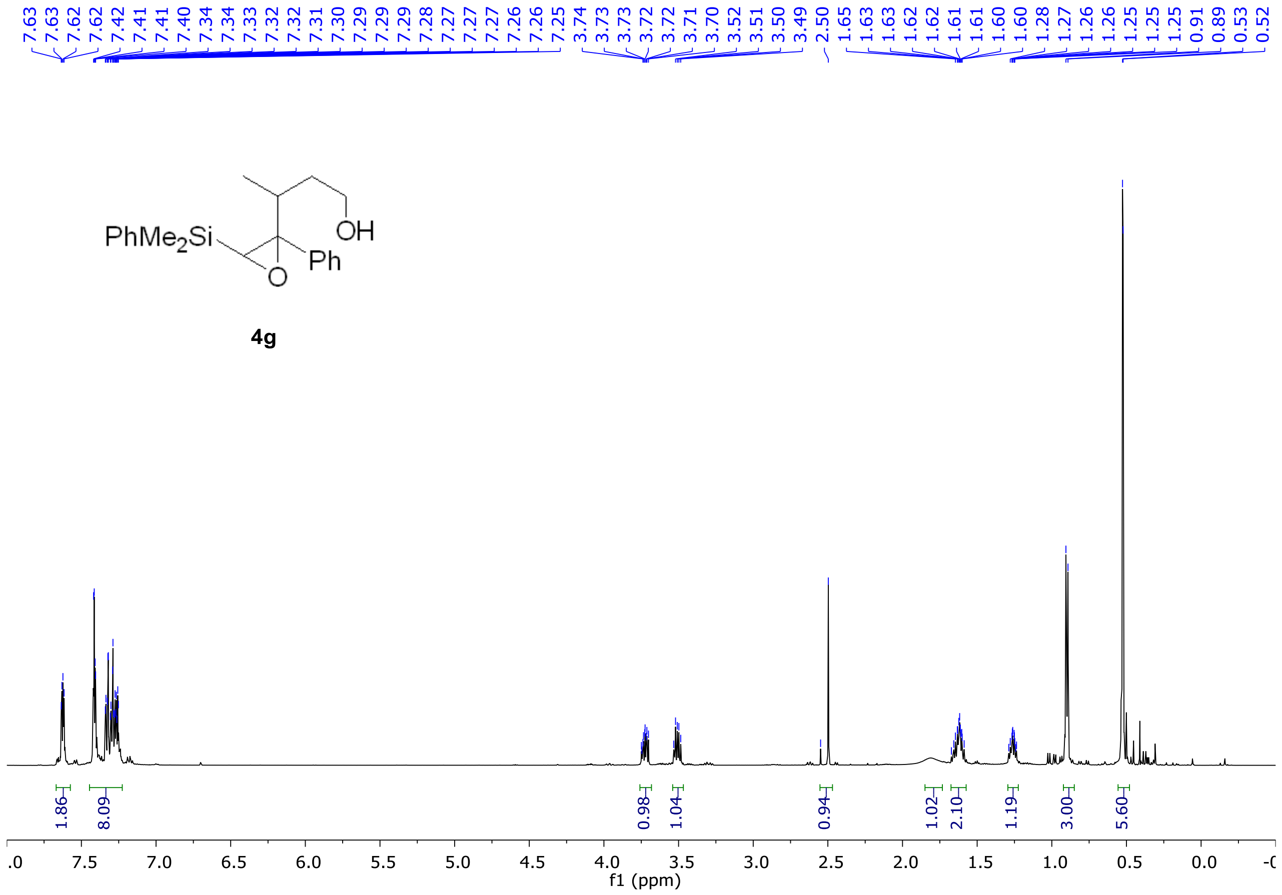

^{13}C NMR (101 MHz, CDCl3)

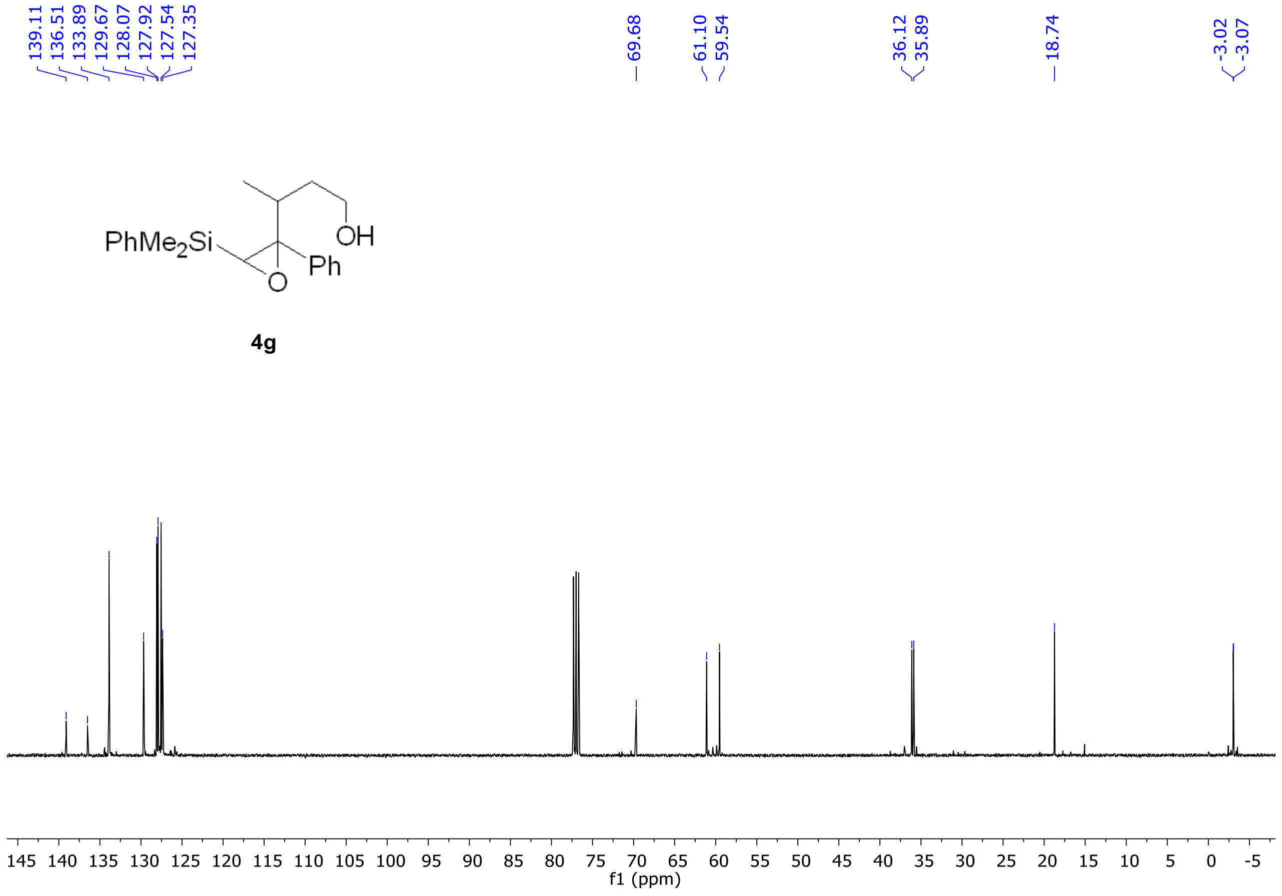

^{1}H NMR (400 MHz, $CDCl_3$)

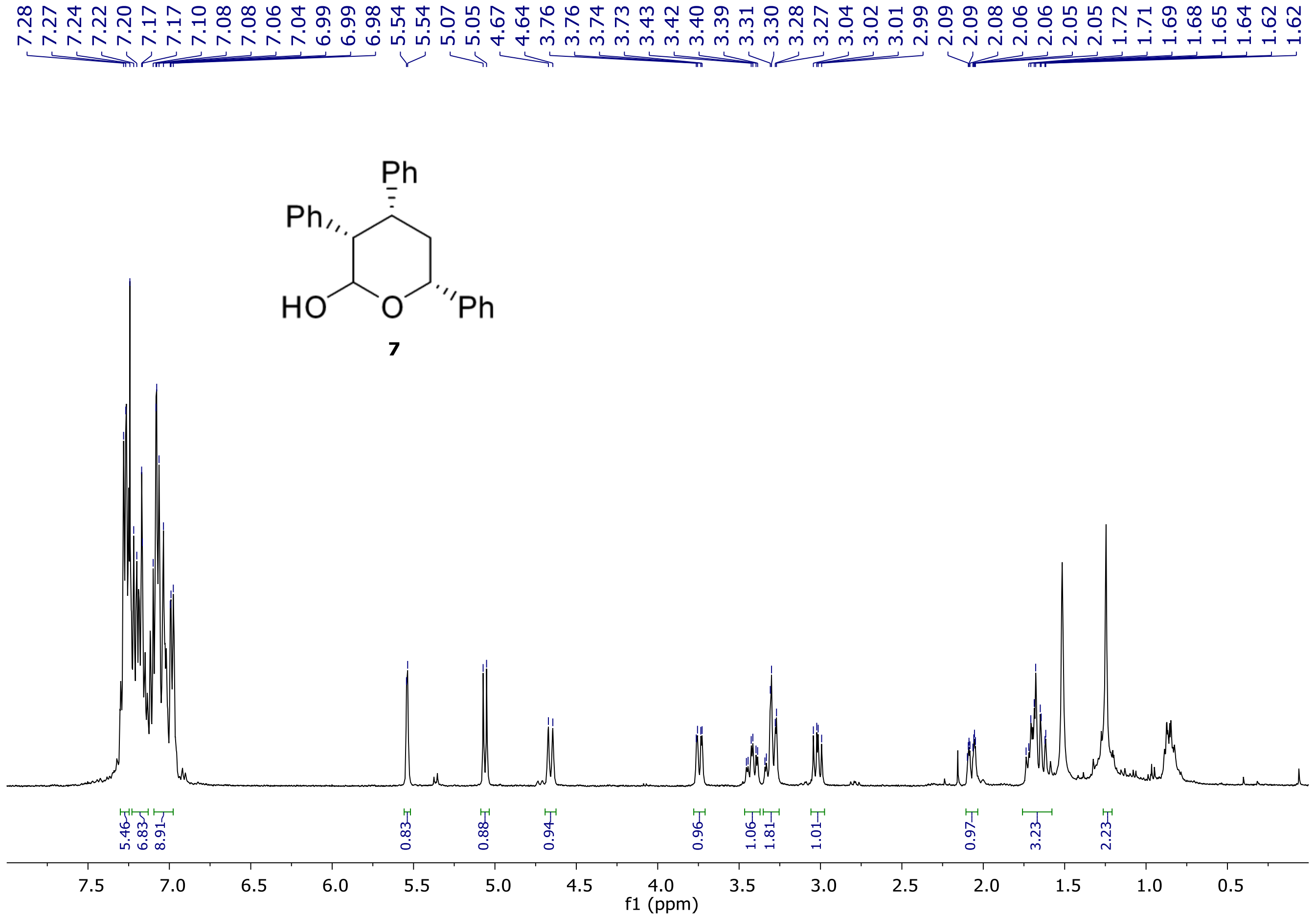

^{13}C NMR (101 MHz, $CDCl_3$)

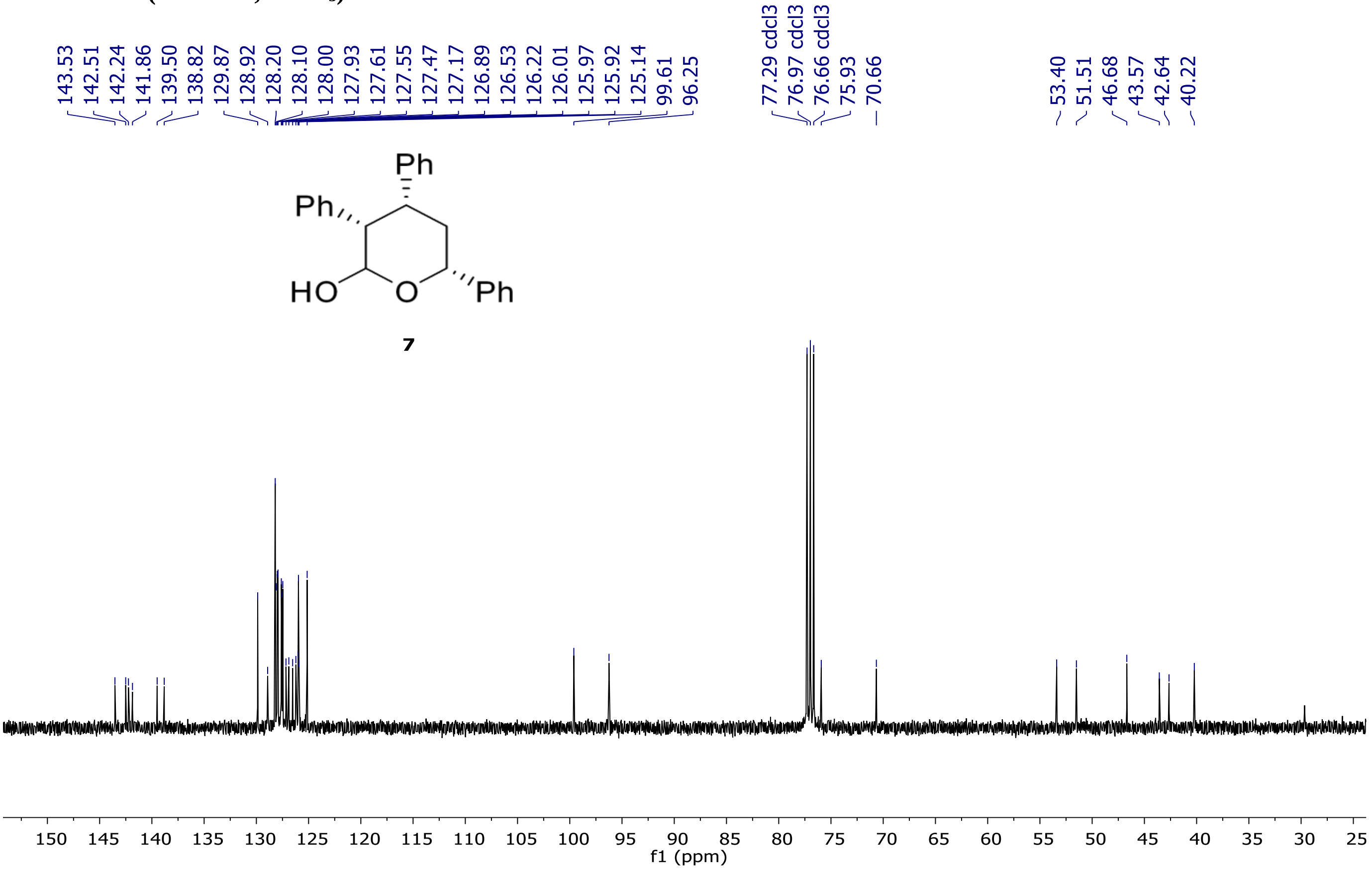

^{1}H NMR (400 MHz, $CDCl_3$)

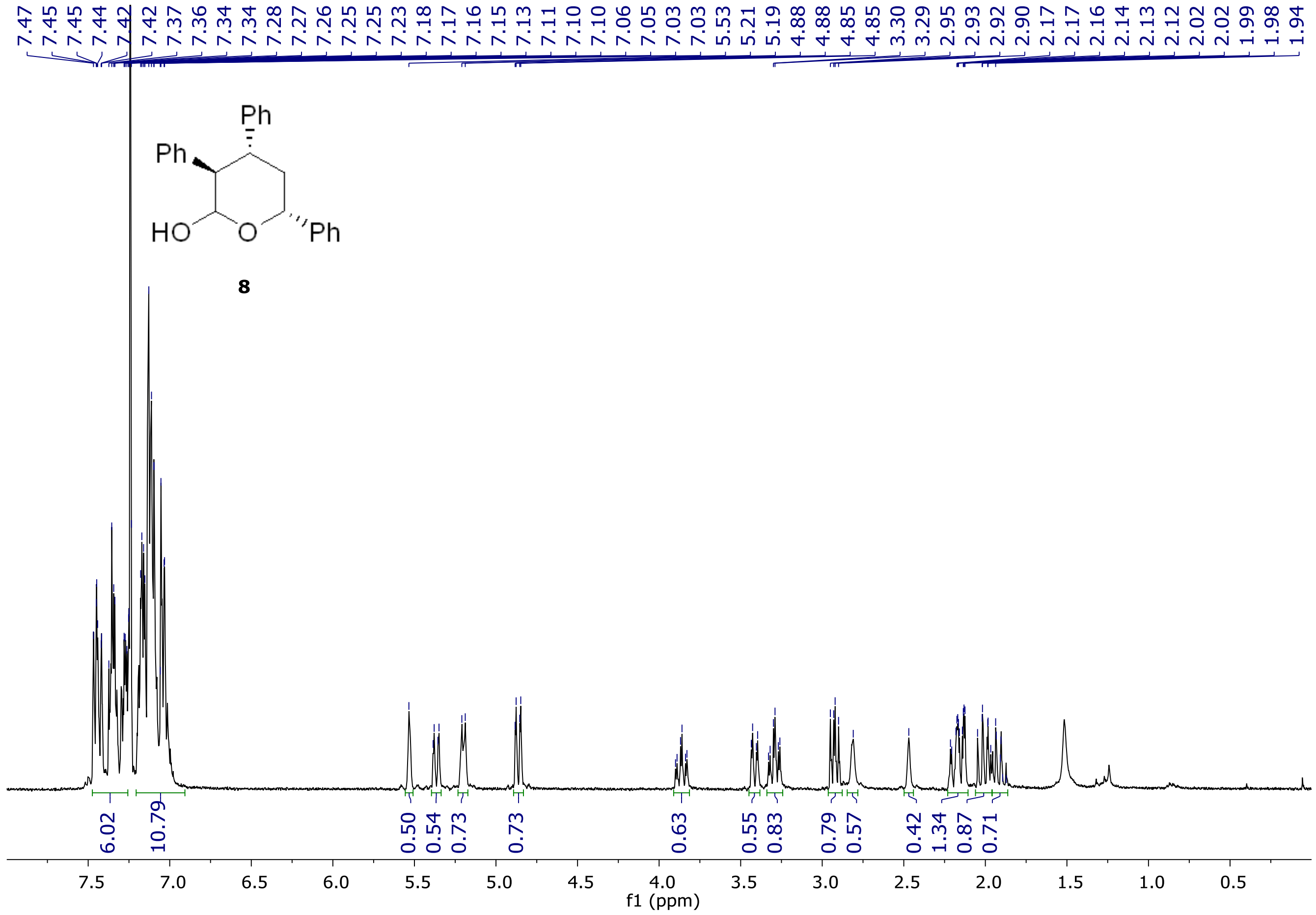

^{13}C NMR (101 MHz, $CDCl_3$)

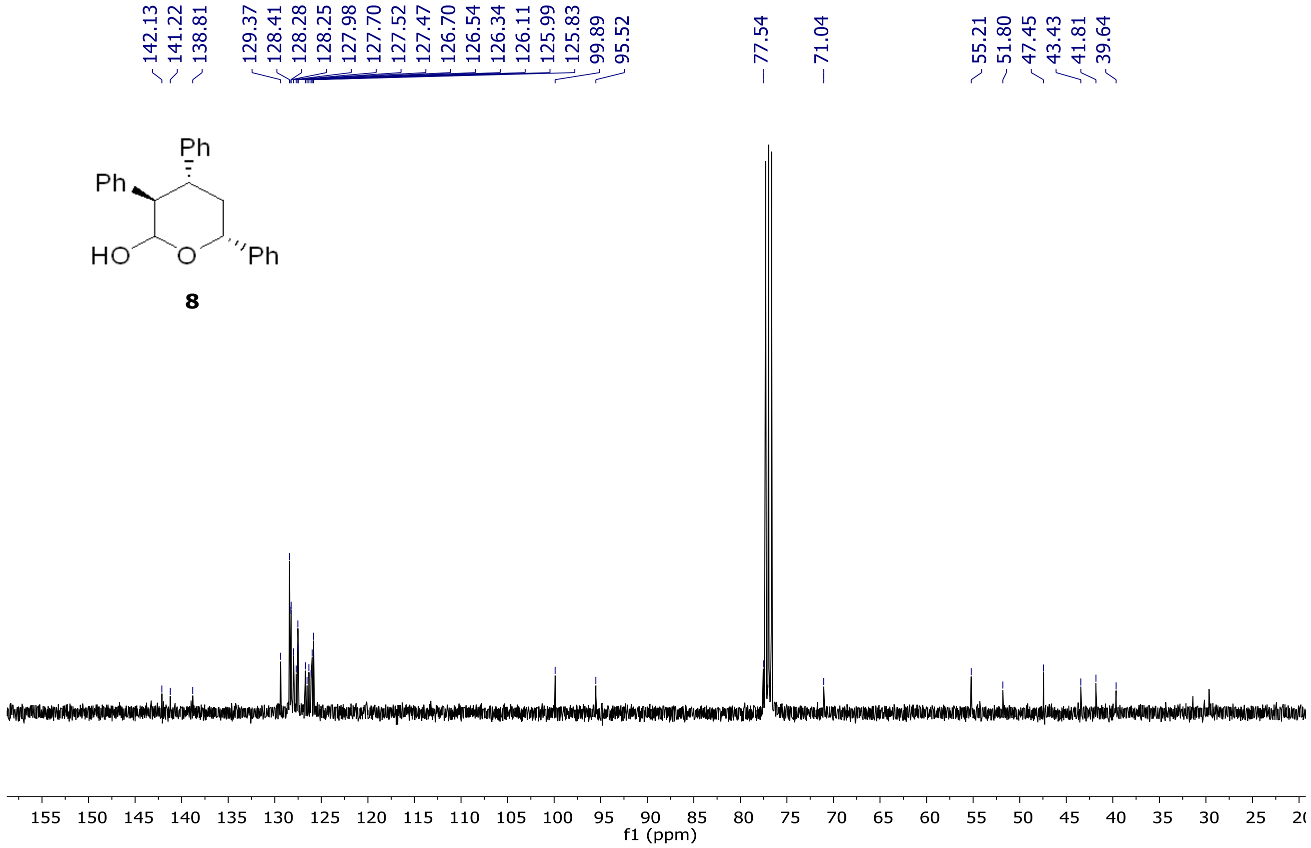

^{1}H NMR (400 MHz, $CDCl_3$)

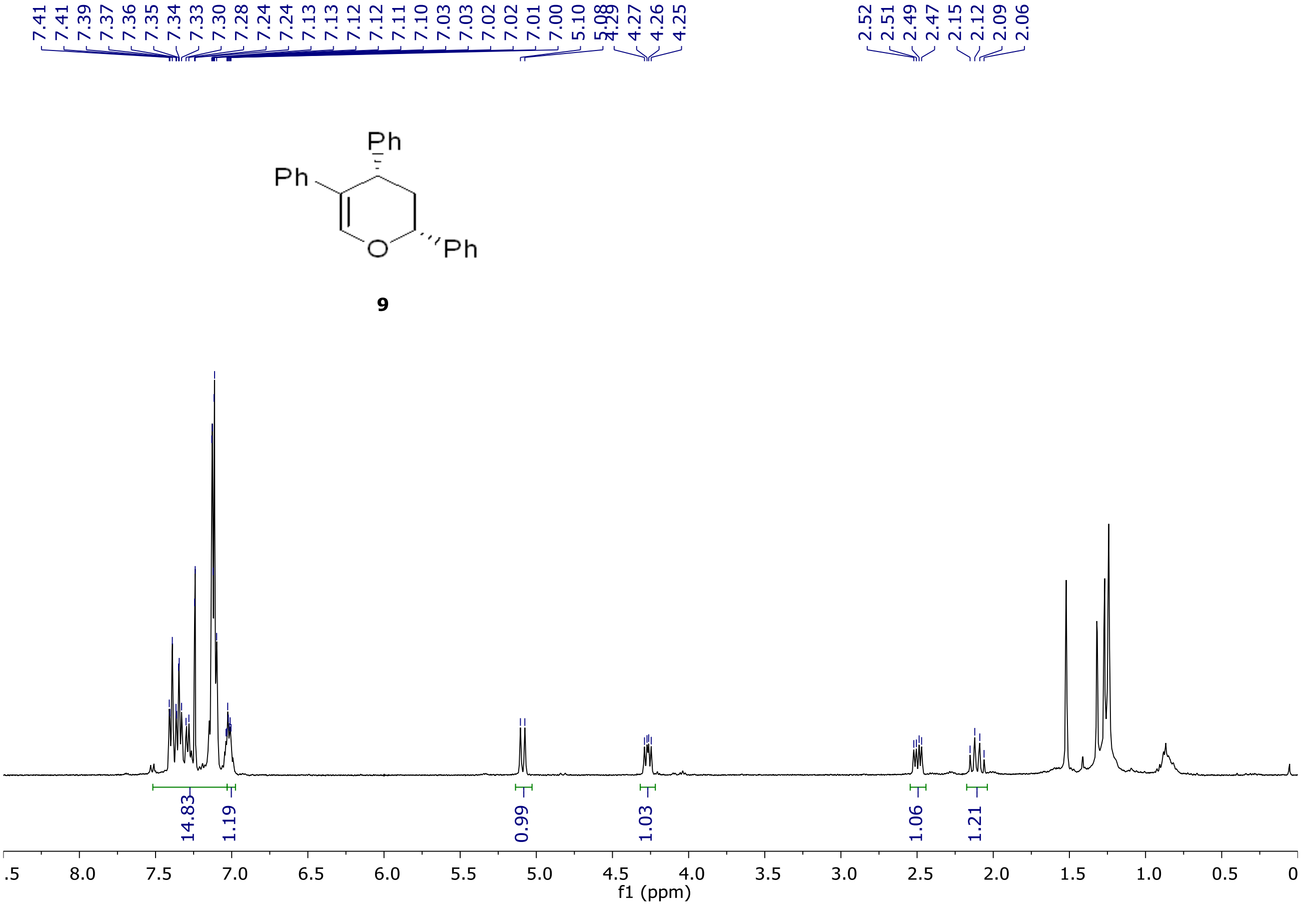

^{13}C NMR (101 MHz, $CDCl_3$)

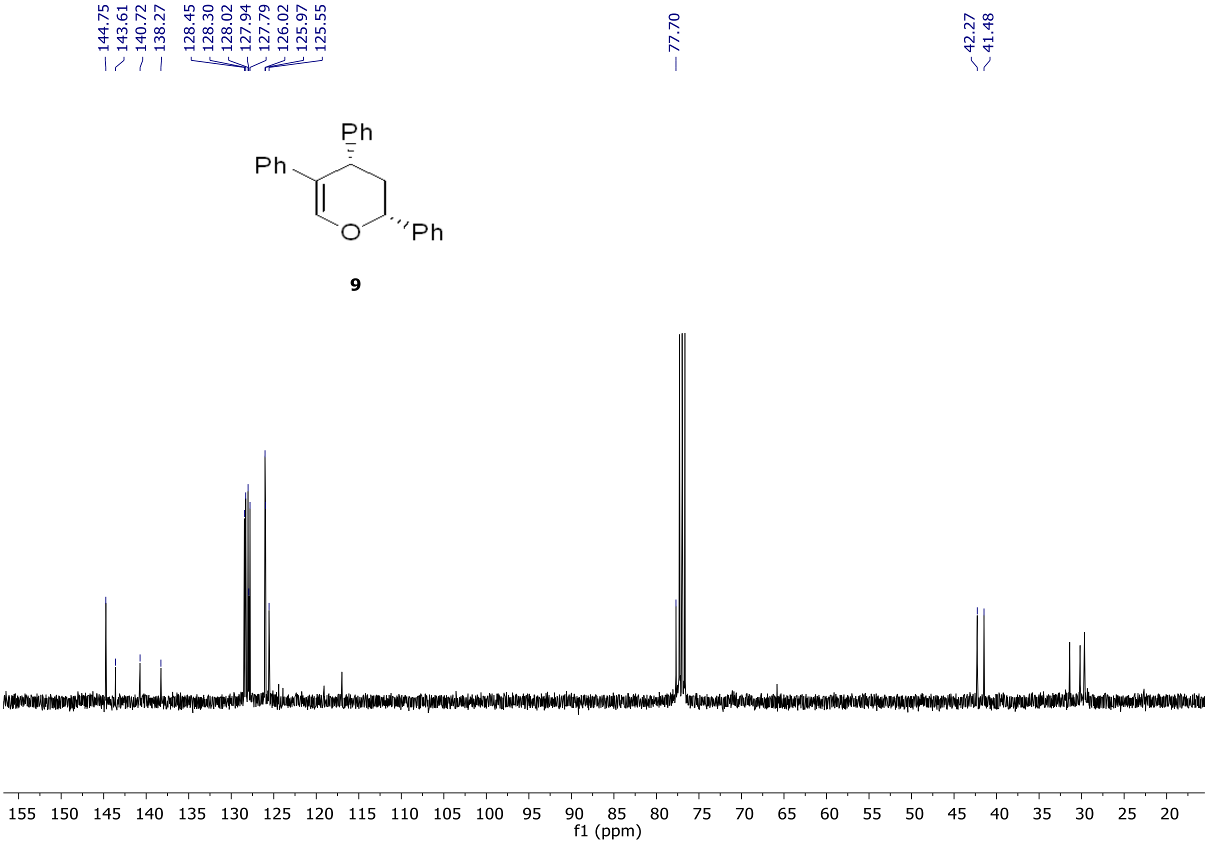

^{1}H NMR (400 MHz, $CDCl_3$)

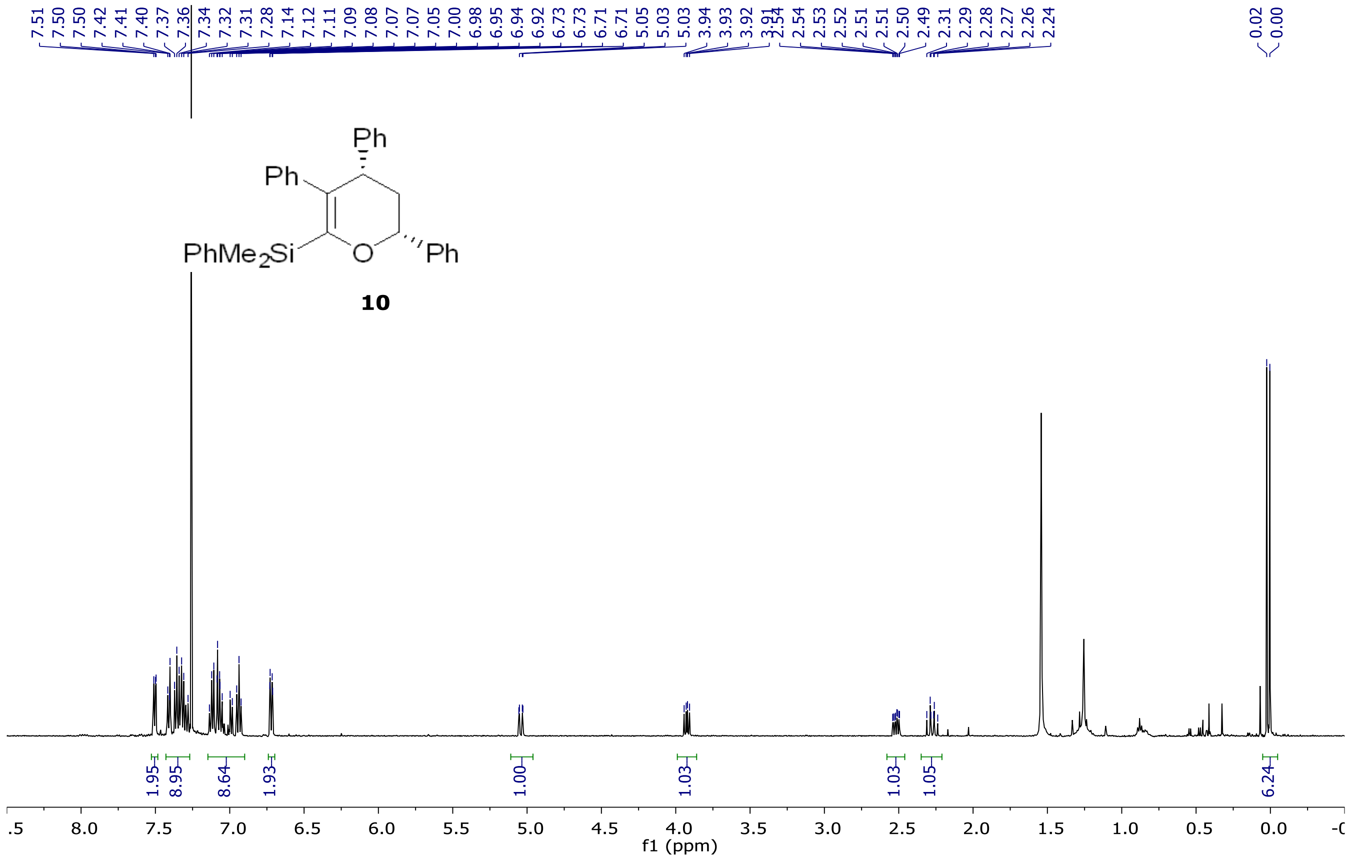

^{13}C NMR (101 MHz, $CDCl_3$)

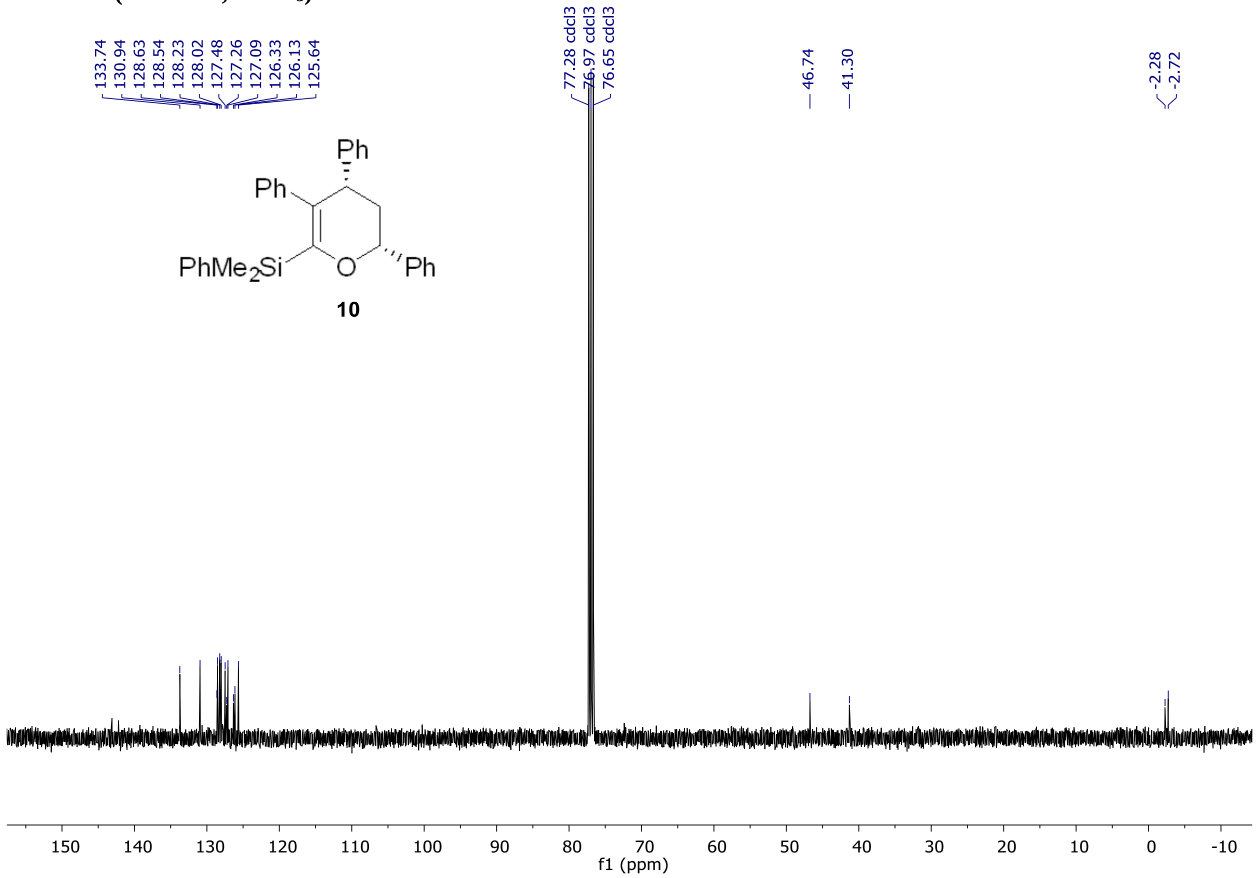

Supplement: Supplementary file 1 [file molecules-26-07386-s001.zip › molecules-1489323-supplementary.pdf]
